# Defining and assessing context in healthcare implementation studies: a systematic review

**DOI:** 10.1186/s12913-020-05212-7

**Published:** 2020-06-29

**Authors:** L. Rogers, A. De Brún, E. McAuliffe

**Affiliations:** 1 University College Dublin Centre for Interdisciplinary Research, Education and Innovation in Health Systems (UCD IRIS), Belfield, Dublin 4, Ireland; 2 University College Dublin School of Nursing, Midwifery and Health Systems, Belfield, Dublin 4, Ireland

**Keywords:** Context, Contextual factors, Healthcare, Implementation science, Systematic review

## Abstract

**Background:**

The implementation of evidence-based healthcare interventions is challenging, with a 17-year gap identified between the generation of evidence and its implementation in routine practice. Although contextual factors such as culture and leadership are strong influences for successful implementation, context remains poorly understood, with a lack of consensus regarding how it should be defined and captured within research. This study addresses this issue by providing insight into how context is defined and assessed within healthcare implementation science literature and develops a definition to enable effective measurement of context.

**Methods:**

Medline, PsychInfo, CINAHL and EMBASE were searched. Articles were included if studies were empirical and evaluated context during the implementation of a healthcare initiative. These English language articles were published in the previous 10 years and included a definition and assessment of context. Results were synthesised using a narrative approach.

**Results:**

Three thousand and twenty-one search records were obtained of which 64 met the eligibility criteria and were included in the review. Studies used a variety of definitions in terms of the level of detail and explanation provided. Some listed contextual factors (*n* = 19) while others documented sub-elements of a framework that included context (n = 19). The remaining studies provide a rich definition of general context (*n* = 11) or aspects of context (*n* = 15). The Alberta Context Tool was the most frequently used quantitative measure (*n* = 4), while qualitative papers used a range of frameworks to evaluate context. Mixed methods studies used diverse approaches; some used frameworks to inform the methods chosen while others used quantitative measures to inform qualitative data collection. Most studies (*n* = 50) applied the chosen measure to all aspects of study design with a majority analysing context at an individual level (*n* = 29).

**Conclusions:**

This review highlighted inconsistencies in defining and measuring context which emphasised the need to develop an operational definition. By providing this consensus, improvements in implementation processes may result, as a common understanding will help researchers to appropriately account for context in research.

## Background

*“Context is one of those words you will encounter again and again, without anyone offering anything like a useful definition. It is something of a catch-all word usually used to mean “all those things in {a} situation which are relevant to the meaning in some sense, but which I haven’t identified”* [1].

The implementation of evidence-based healthcare interventions is challenging with a 17-year gap identified between the generation of evidence and the implementation of interventions in routine practice [2–5]. It is argued that we cannot understand or explain these findings without looking at the context in which the intervention was embedded [6–8]. However, contextual factors are often overlooked by researchers in the field of implementation science. Despite its proclaimed influence on implementation efforts [9–12], context remains poorly understood and reported [13]. This insufficient understanding has been attributed to the variability in how context is defined and measured in various studies [13–16]. An example of this lack of consensus is reflected in the terms used within implementation science frameworks. Few refer to context explicitly (e.g. Promoting Action on Research Implementation in Health Services (PARiHS)) with a majority referring to the construct indirectly using expressions such as “inner setting” and “outer setting” (Consolidated Framework for Implementation Research (CFIR)).

Research that has begun to investigate how context is conceptualised have confirmed the existence of inconsistencies [16–18]. McCormack et al. [17] was one of the first to complete a concept analysis of ‘context’. They reported that the lack of clarity associated with context was related to its characterisation as an objective entity; the environment or setting in which the intervention is implemented. Similarly, Pfadenhauer et al. [16] found that the construct remains unmatured but suggest that instead of a passive phenomenon, context is dynamic, embracing not only the physical setting but also the social environment. While writing the findings of this review article, a scoping review by Nilsen and Bernhardsson [18] was published investigating the conceptualisation of context within determinant frameworks. Like the aforementioned studies [16, 17], Nilsen and Bernhardsson found that most studies included a narrow description of context but they also identified common contextual determinants across frameworks including; organisational support, financial resources, social relations, leadership, and organisational culture and climate. Although Nilsen and Bernhardsson’s article conceptualises context, the findings are limited to definitions provided within the determinant frameworks of the 22 included publications. Therefore, the question remains as to how context is defined more broadly within implementation research and whether the determinants identified by Nilsen and Bernhardsson are applicable to this wider literature base. This review addresses this question and aims to improve the consistency for the use of the term ‘context’ by developing an operational definition for this construct.

Literature suggests that to understand the dynamic relationship between context and implementation, conceptualisations of context need to be translated into practical methods of assessment [19, 20]. Fernandez et al. [20] argue that the ability to intervene upon contextual factors is dependent on an ability to measure them. However, the subordinate role context plays within implementation research [16] has led to a dearth of guidance on how to measure it. Lewis et al. [21] identified 420 instruments relevant to implementation science, however, it is unclear what methods exist to specifically measure contextual domains. Additionally it is recognised that interactions between context and intervention implementation occurs at multiple levels of the system [18], something that has not been examined in existing literature.

To address identified gaps in the evidence base, this paper aims to answer the following research question: “How is context defined and measured within healthcare implementation science literature?” Whilst providing greater clarity regarding how context is defined, assessed and analysed, it is hoped this review will also enhance the rigour of future studies exploring context within implementation research. The development of an operational definition and the identification of methods of assessment may better enable comparative evaluations to be conducted, enhancing our understanding of how context influences implementation processes.

## Methods

This systematic review was conducted to explore the proposed research question. This study was informed by the Cochrane handbook’s [22] guidance for conducting systematic reviews and the Preferred Reporting Items of Systematic Reviews and Meta-Analyses (PRISMA) [23]. The review protocol was published on the PROSPERO Database in January 2019 (CRD42019110922).

### Search strategy

Extant literature within the field of implementation science informed the search strategy (Additional file 1). Using keywords in conjunction with truncation and Boolean operators, four electronic databases were searched: Medline, CINAHL, EMBASE and PsychINFO. Reference lists of included studies were also hand searched to identify potentially relevant studies that were not retrieved from the database searches. However, no additional relevant articles were retrieved.

### Inclusion and exclusion criteria

The studies were restricted to peer-reviewed articles published in English in the previous 10 years (January 1st, 2008 to September 25th, 2018). The eligibility criteria were broad to ensure a balance between a specific and sensitive search of the literature. Empirical studies were included if context was a key component or focus during the implementation of an initiative in a healthcare setting and if a definition and assessment of context was included.

### Study screening and data extraction

Covidence [24], an online data management system, was employed to manage the review process. Article screening and selection was performed independently by two reviewers (LR and ADB) against the eligibility criteria. Any disagreements over a study’s inclusion was resolved through discussion with a third reviewer (EMC) (*n* = 2). To guide data abstraction, the reviewers developed a standardised data extraction tool (Additional file 2). The quality of the definition outlined in each article was assessed by identifying whether articles simply listed contextual factors relating to the construct, outlined sub-elements of a framework that included context or if a rich definition of context was provided (Table 1). Kirk et al.’s [29] approach was adopted to examine the depth of application of each context assessment by determining 1) how the method was used in the included studies (data collection, descriptive data analysis, or both); 2) whether the measure was used to investigate the association between context and implementation success; and 3) examine the unit of analysis (individual, team, organisation, system level).

**Table 1:** Description of definition quality

Definition type	Description	Example
Lists contextual factors	Articles list contextual factors relevant to the implementation process	*“Management support, adequate training, physician involvement, physician autonomy and doctor-patient relationship”* [﻿25﻿]
Lists sub-elements of a framework that includes context	Articles list sub-elements of a framework that includes contextual factors	“*Context includes aspects of the culture, leadership and evaluation at the particular site”* *(Definition based on the PARiHS framework)* [﻿26﻿].
Provides a meaningful definition of general context	Articles go beyond simply listing contextual factors and provide a broad definition of context	“*… the pre-existing conditions and relationships in the organisational system that partner with the programme’s mechanisms to make success or failure of the intervention more or less likely”* [﻿27﻿].
Provides a meaningful definition of an element of context	Articles provide a rich definition of an aspect of context or provide a comprehensive definition of sub-elements of a framework that includes context	“*Organisational climate is conceptualised as individual and group perceptions of how the work environment affects the well-being of the organisations members while organisational culture is conceptualised as how the work is done in the organisation based on worker expectations”* [﻿28﻿].

### Quality appraisal

As appropriate for the study design, sections of the Mixed Methods Appraisal Tool (MMAT) were used to assess the quality of each included article [30]. Consistent with best practice, two reviewers (LR and ADB) independently appraised each study. Any disagreements over the quality or risk of bias of included papers were resolved through discussion. To enhance the transparency in reporting the appraisal process, a summary of the quality assessment can be seen in Table 2.

**Table 2:** Summary of Quality Appraisal

***2A Quality appraisal of qualitative articles and the qualitative aspects of mixed methods studies***					
**Author, Year**	**Qualitative approach appropriate?**	**Qualitative data collection methods adequate?**	**Findings adequately derived from the data?**	**Is the interpretation of results sufficiently substantiated by data?**	**Coherence between qualitative data sources, collection, analysis and interpretation?**
Al Shemeili et al. (2016) [31]	Yes	Yes	Yes	Yes	Yes
Arney et al. (2018) [32]	Yes	Yes	Yes	Yes	Yes
Bain et al. (2015) [33]	Yes	Yes	Yes	Yes	Yes
Beidas et al. (2014) (Mixed methods) [34]	Yes	Yes	Yes	Yes	Yes
Belaid & Ridde (2015) [35]	Yes	Yes	Yes	Yes	Yes
Bergström et al. (2012) [36]	Yes	Yes	Yes	Yes	Yes
Bocoum et al. (2017) [37]	Yes	Yes	Yes	Yes	Yes
Bokhour et al. (2015) [26]	Yes	Yes	Yes	Yes	Yes
Bradley & Griffin (2016) (Mixed methods) [27]	Yes	Yes	Yes	Yes	Yes
Burau et al. (2018) [38]	Yes	Yes	Yes	Yes	Yes
Busetto et al. (2017) [39]	Yes	Yes	Yes	Yes	Yes
Cheyne et al. (2013) [40]	Yes	Yes	Yes	Yes	Yes
Drainoni et al. (2016) [41]	Yes	Yes	Yes	Yes	Yes
Eboreime et al. (2018) (Mixed methods) [42]	Yes	Yes	Yes	Yes	Yes
Gadomski et al. (2014) [43]	Yes	Yes	Yes	Yes	Yes
Gagliardi et al. (2014) [44]	Yes	Yes	Yes	Yes	Yes
Georgiou & Westbrook (2009) [45]	Yes	Yes	Yes	Yes	Yes
Gibb (2013) (Mixed-methods) [46]	Yes	Yes	Yes	Yes	Yes
Glidewell et al. (2013) [47]	Yes	Yes	Yes	Yes	Yes
Greenhalgh et al. (2008) (Mixed methods) [48]	Yes	Yes	Yes	Yes	Yes
Griffin et al. (2017) [49]	Yes	Yes	Yes	Yes	Yes
Hansen et al. (2011) [8]	Yes	Yes	Yes	Yes	Yes
Higgins et al. (2015) [50]	Yes	Yes	Yes	Yes	Yes
Kramer et al. (2017) (Mixed methods) [28]	Yes	Yes	Yes	Yes	Yes
Menon et al. (2014) (Mixed methods) [51]	Yes	Yes	Yes	Yes	Yes
Murdoch (2016) [19]	Yes	Yes	Yes	Yes	Yes
Naik et al. (2015) [52]	Yes	Yes	Yes	Yes	Yes
Padwa et al. (2016) (Mixed-methods) [53]	Yes	Yes	Yes	Yes	Yes
Presseau et al. (2017) [54]	Yes	Yes	Yes	Yes	Yes
Rabbani et al. (2011) (Mixed methods) [55]	Yes	Yes	Yes	Yes	Yes
Rotteau et al. (2015) [56]	Yes	Yes	Yes	Yes	Yes
Smith et al. (2018) [57]	Yes	Yes	Yes	Yes	Yes
Spitzer-Shohat et al. (2018 (Mixed methods) [58]	Yes	Yes	Yes	Yes	Yes
VanDevanter et al. (2017) [59]	Yes	Yes	Yes	Yes	Yes
Ware et al. (2018) [60]	Yes	Yes	Yes	Yes	Yes
Williams et al (2016) [61]	Yes	Yes	Yes	Yes	Yes
Yamada et al. (2018) [62]	Yes	Yes	Yes	Yes	Yes
Yip et al. (2016) [63]	Yes	Yes	Yes	Yes	Yes
Durbin et al. (2016) [64]	Yes	Yes	Yes	Can’t tell	Yes
Erasmus et al. (2017) (Mixed methods) [65]	Yes	Yes	Yes	Can’t tell	Yes
Hill et al. (2017) [66]	Yes	Yes	Yes	Can’t tell	Yes
Iribarren et al (2015) [67]	Yes	Yes	Yes	Can’t tell	Can’t tell
Prashanth et al. (2014) [68]	Yes	Yes	Can’t tell	Can’t tell	Can’t tell
Rodríguez & Peterson (2016) [69]	Yes	Can’t tell	Can’t tell	Can’t tell	Can’t tell
Baron & Newman (2016) (Mixed methods) [70]	Yes	Can’t tell	Can’t tell	Can’t tell	Can’t tell
Vanderkruik & McPherson (2017) [71]	Yes	Can’t tell	Can’t tell	Can’t tell	Can’t tell
***2B Quality appraisal of randomised control trials and quantitative aspects of a mixed method study***					
**Author, Year**	**Randomization appropriately performed?**	**Are the groups comparable at baseline?**	**Are there complete outcome data?**	**Are outcome assessors blinded to the intervention?**	**Did participants adhere to the assigned intervention?**
Forberg et al. (2016) [72]	Yes	Yes	Yes	Can’t tell	Yes
Chan et al. (2011) [73]	Can’t tell	Can’t tell	Yes	Can’t tell	Can’t tell
Baron & Newman (2016) (Mixed methods) [70]	No	Yes	No	Can’t tell	Yes
***2C Quality appraisal of non-randomised control trial***					
**Author, Year**	**Sample representative?**	**Measurements appropriate?**	**Complete outcome data?**	**Confounders accounted for?**	**Intervention administered (or exposure occurred) as intended?**
Abdekhoda et al. (2015) [25]	Yes	Yes	Yes	Can’t tell	Yes
***2D Quality appraisal of quantitative articles and the quantitative aspects of mixed methods studies***					
**Author, Year**	**Sampling strategy relevant?**	**Sample representative?**	**Measurements appropriate?**	**Risk of nonresponse bias low?**	**Statistical analysis appropriate?**
Beenstock et al. (2012) [74]	Yes	Yes	Yes	Yes	Yes
Cummings et al. (2010) [75]	Yes	Yes	Yes	Yes	Yes
Glisson et al. (2008) [76]	Yes	Yes	Yes	Yes	Yes
Guerrero et al. (2015) [77]	Yes	Yes	Yes	Yes	Yes
Hoffman & Rodrígrez (2015) [78]	Yes	Yes	Yes	Yes	Yes
Lemmens et al. (2009) [79]	Yes	Yes	Yes	Yes	Yes
Eboreime et al. (2018) (Mixed methods) [42]	Yes	N/A	Yes	N/A	N/A
Abdekhoda et al. (2015) [25]	Yes	Yes	Yes	Can’t tell	Yes
Almblad et al. (2018) [80]	Yes	Yes	Yes	Can’t tell	Yes
Beidas et al. (2014) (Mixed methods) [34]	Yes	Yes	Yes	Can’t tell	Yes
Bradley & Griffin (2016) (Mixed methods) [27]	Yes	Yes	Yes	Can’t tell	Yes
Chiu & Ku (2015) [81]	Yes	Yes	Yes	Can’t tell	Yes
Ehrhart et al. (2014) [82]	Yes	Yes	Yes	Can’t tell	Yes
Erasmus et al. (2017) (Mixed methods) [65]	Yes	Yes	Yes	Can’t tell	Yes
Huijg et al. (2014) [83]	Yes	Yes	Yes	Can’t tell	Yes
Fernandez et al. (2018) [20]	Yes	Yes	Yes	Can’t tell	Yes
Kramer et al. (2017) (Mixed methods) [28]	Yes	Can’t tell	Yes	Yes	Yes
Menon et al. (2014) (Mixed methods) [51]	Yes	Yes	Yes	Can’t tell	Yes
Obrecht et al. (2014) [84]	Yes	Yes	Yes	Can’t tell	Yes
Rabbini et al. (2011) (Mixed methods) [55]	Yes	Yes	Yes	Can’t tell	Yes
Spitzer-Shohat et al. (2018) (Mixed methods) [58]	Yes	Yes	Yes	Can’t tell	Yes
Greenhalgh et al. (2008) (Mixed methods) [48]	Yes	Can’t tell	Yes	Yes	N/A
Beidas et al. (2015) [85]	Yes	Yes	Yes	Can’t tell	Yes
Douglas (2016) [86]	Yes	Can’t tell	Yes	No	Yes
Padwa et al. (2016) (Mixed methods) [53]	Yes	Can’t tell	Yes	Can’t tell	Yes
Yamada et al. (2017) [87]	Yes	Can’t tell	Yes	Can’t tell	Yes
Gibb (2013) (Mixed methods) [46]	Can’t tell	Can’t tell	Can’t tell	Can’t tell	Can’t tell
***2E Quality appraisal of mixed-methods studies***					
**Author, Year**	**Adequate rationale for using a mixed methods design?**	**Different components of the study effectively integrated?**	**Outputs of the integration of qualitative and quantitative components adequately interpreted?**	**Divergences and inconsistencies between quantitative and qualitative results adequately addressed?**	**Different components of the study adhere to the quality criteria of each tradition of the methods involved?**
Beidas et al. (2014) [34]	Yes	Yes	Yes	Yes	Yes
Bradley & Griffin (2016) [27]	Yes	Yes	Yes	Yes	Yes
Kramer et al. (2017) [28]	Yes	Yes	Yes	Yes	Yes
Rabbini et al. (2011) [55]	Yes	Yes	Yes	Yes	Yes
Spitzer-Shohat et al. (2018) [58]	Yes	Yes	Yes	Yes	Yes
Eboreime et al. (2018) [42]	Yes	Yes	Yes	Can’t tell	Yes
Erasmus et al. (2017) [65]	Yes	Yes	Yes	Can’t tell	Yes
Greenhalgh et al. (2008) [48]	Yes	Yes	Yes	Can’t tell	Yes
Menon et al. (2014) [51]	Yes	Yes	Yes	Can’t tell	Yes
Padwa et al. (2016) [53]	Yes	Yes	Yes	Can’t tell	Yes
Gibb (2013) [46]	Yes	Yes	Yes	Can’t tell	Can’t tell
Baron & Newman (2016) [70]	Can’t tell	Yes	Yes	Can’t tell	Can’t tell

### Data synthesis

Due to aim of this study and the heterogeneity of the articles included, a narrative synthesis [88] and thematic analysis [89] of the findings was the most appropriate approach to examine the review question. This approach enabled the extent of convergence, divergence, and contradiction among studies on how to define, and assess context to be explored [90]. NVivo software was used to manage the synthesis of the data and the results are reported in accordance with PRISMA guidelines [23].

## Results

**Fig. 1:**
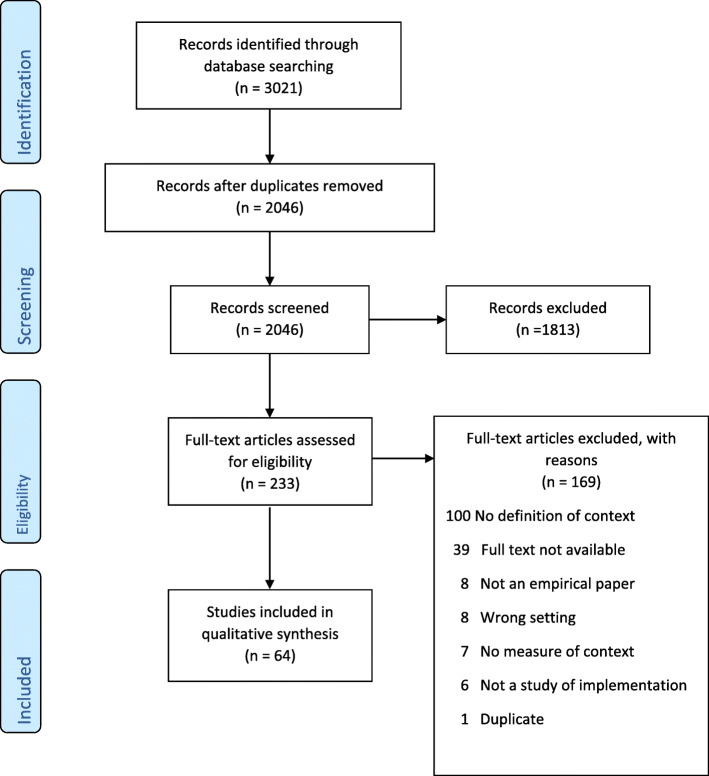
PRISMA flow diagram

The search returned a total of 3021 records. Of these, 975 were duplicates and were removed. One thousand, eight hundred, and thirteen articles were excluded following title and abstract screening and a further 169 were excluded during full text review as they did not meet the inclusion criteria. Reasons for exclusion are evident on the PRISMA diagram (Fig. 1). On further assessment 60 of these papers had more than one reason for exclusion. In total 64 studies met the inclusion criteria and were reviewed.

Table S1 and Table 3 summarise the studies included in this review, outlining the study characteristics, the quality of the definitions employed, the depth of application for these measures and the unit of analysis chosen by each study.

**Table 3:** Summary table-quality of definition, application of context measure, unit of analysis

Article citation	Methodology	Quality of definition	Data collection	Descriptive analysis	Investigate association between context and implementation success
**Unit of analysis-Individual**					
Abdekhoda et al. (2015) [25]	Quantitative	Lists contextual factors	✓	✓	✓
Al Shemeili et al. (2016) [31]	Qualitative	Provides a meaningful definition of an element of context	✓	✓	
Arney et al. (2018) [32]	Qualitative	Lists sub-elements of a framework that includes context	✓	✓	✓
Bain et al. (2015) [33]	Qualitative	Provides a meaningful definition of an element of context	✓	✓	✓
Beenstock et al. (2012) [74]	Quantitative	Provides a meaningful definition of an element of context	✓	✓	✓
Bokhour et al. (2015) [26]	Qualitative	Lists sub-elements of a framework that includes context		✓	✓
Chiu & Ku (2015) [81]	Quantitative	Lists contextual factors		✓	✓
Douglas (2016) [86]	Quantitative	Lists sub-elements of a framework that includes context	✓	✓	✓
Drainoni et al. (2016) [41]	Qualitative	Lists sub-elements of a framework that includes context	✓	✓	✓
Förberg et al. (2016) [72]	Quantitative	Lists sub-elements of a framework that includes context	✓	✓	✓
Gadomski et al. (2014) [43]	Qualitative	Lists sub-elements of a framework that includes context	✓	✓	✓
Gagliardi et al. (2014) [﻿44﻿]	Qualitative	Provides a meaningful definition of an element of context		✓	✓
Georgiou & Westbrook (2009) [45]	Qualitative	Lists sub-elements of a framework that includes context	✓	✓	✓
Gibb (2013) [46]	Mixed methods	Lists sub-elements of a framework that includes context	✓	✓	✓
Glidewell et al. (2013) [47]	Qualitative	Provides a meaningful definition of an element of context	✓	✓	✓
Glisson et al. (2008) [76]	Quantitative	Provides a meaningful definition of an element of context	✓	✓	✓
Huijg et al. (2014) [83]	Quantitative	Lists contextual factors	✓	✓	✓
Lemmens et al. (2009) [79]	Quantitative	Lists sub-elements of a framework that includes context	✓	✓	✓
Naik et al. (2015) [52]	Qualitative	Lists sub-elements of a framework that includes context	✓	✓	✓
Obrecht et al. (2014) [84]	Quantitative	Provides a meaningful definition of an element of context	✓	✓	✓
Prashanth et al. (2014) [68]	Qualitative	Provides a meaningful definition of general context	✓	✓	✓
Presseau et al. (2017) [54]	Qualitative	Lists sub-elements of a framework that includes context	✓	✓	✓
Rodríguez & Peterson (2016) [69]	Qualitative	Lists contextual factors	✓	✓	✓
Rotteau et al. (2015) [56]	Qualitative	Lists contextual factors	✓		✓
Smith et al. (2018) [57]	Qualitative	Provides a meaningful definition of an element of context	✓	✓	✓
VanDevanter et al. (2017) [59]	Qualitative	Lists sub-elements of a framework that includes context	✓	✓	✓
Ware et al. (2018) [60]	Qualitative	Lists sub-elements of a framework that includes context	✓	✓	✓
Yamada et al. (2017) [87]	Quantitative	Lists contextual factors	✓	✓	✓
Yamada et al. (2018) [62]	Qualitative	Provides a meaningful definition of an element of context	✓	✓	✓
**Unit of analysis-Individual & Team**					
Cummings et al. (2010) [75]	Quantitative	Lists sub-elements of a framework that includes context	✓	✓	✓
Ehrhart et al. (2014) [82]	Quantitative	Provides a meaningful definition of an element of context	✓	✓	✓
**Unit of analysis-Team**					
Almblad et al. (2018) [80]	Quantitative	Lists contextual factors	✓	✓	✓
Fernandez et al. (2018) [20]	Quantitative	Provides a meaningful definition of an element of context	✓	✓	✓
Vanderkruik & McPherson (2017) [71]	Qualitative	Provides a meaningful definition of general context	✓	✓	✓
**Unit of analysis-Project**					
Hill et al. (2017) [66]	Qualitative	Lists sub-elements of a framework that includes context		✓	✓
**Unit of analysis-Organisational**					
Burau et al. (2018) [38]	Qualitative	Provides a meaningful definition of general context	✓	✓	✓
Busetto et al. (2017) [39]	Qualitative	Provides a meaningful definition of general context	✓	✓	✓
Cheyne et al. (2013) [40]	Qualitative	Provides a meaningful definition of general context	✓	✓	✓
Durbin et al. (2016) [64]	Qualitative	Lists contextual factors	✓	✓	✓
Erasmus et al. (2017) [65]	Mixed methods	Provides a meaningful definition of an element of context	✓	✓	✓
Guerrero et al. (2015) [77]	Quantitative	Lists contextual factors	✓	✓	✓
Rabbani et al. (2011) [55]	Mixed methods	Provides a meaningful definition of general context	✓		✓
Spitzer-Shohat et al. (2018) [58]	Mixed methods	Lists contextual factors	✓	✓	✓
Williams et al. (2016) [61]	Qualitative	Lists contextual factors	✓	✓	✓
**Unit of analysis-Individual and organisational**					
Beidas et al. (2014) [34]	Mixed methods	Provides a meaningful definition of an element of context	✓	✓	✓
Beidas et al. (2015) [85]	Quantitative	Lists contextual factors	✓	✓	✓
Bergstrom et al. (2012) [36]	Qualitative	Provides a meaningful definition of an element of context	✓	✓	✓
Bocoum et al. (2017) [37]	Qualitative	Lists contextual factors	✓	✓	
Bradley & Griffin (2016) [27]	Mixed methods	Provides a meaningful definition of general context		✓	
Greenhalgh et al. (2008) [48]	Mixed methods	Lists contextual factors	✓		✓
Griffin et al. (2017) [49]	Qualitative	Lists sub-elements of a framework that includes context	✓	✓	✓
Hansen et al. (2011) [8]	Qualitative	Provides a meaningful definition of general context	✓	✓	✓
Higgins et al. (2015) [50]	Qualitative	Lists contextual factors	✓	✓	✓
Iribarren et al. (2015) [67]	Qualitative	Lists sub-elements of a framework that includes context	✓	✓	✓
Kramer et al. (2017) [28]	Mixed methods	Provides a meaningful definition of an element of context	✓	✓	✓
Menon et al. (2014) [51]	Mixed methods	Lists sub-elements of a framework that includes context	✓	✓	✓
Padwa et al. (2016) [53]	Mixed methods	Lists sub-elements of a framework that includes context	✓	✓	✓
**Unit of analysis-Team and organisational**					
Chan et al. (2011) [73]	Quantitative	Lists contextual factors		✓	✓
Eboreime et al. (2014) [42]	Mixed methods	Lists contextual factors	✓	✓	✓
**Unit of analysis-Individual, organisational and regional**					
Hoffman & Rodriguez (2015) [78]	Quantitative	Lists contextual factors		✓	✓
**Unit of analysis-Organisational and regional**					
Belaid & Ridde (2015) [35]	Qualitative	Provides a meaningful definition of general context	✓	✓	✓
**Unit of analysis-Individual, organisational and national**					
Murdoch (2016) [19]	Qualitative	Provides a meaningful definition of general context		✓	✓
Yip et al. (2016) [63]	Qualitative	Lists contextual factors	✓	✓	✓
**Unit of analysis-Unclear**					
Baron & Newman (2016) [70]	Mixed methods	Provides a meaningful definition of general context		✓	✓

**Fig. 2:**
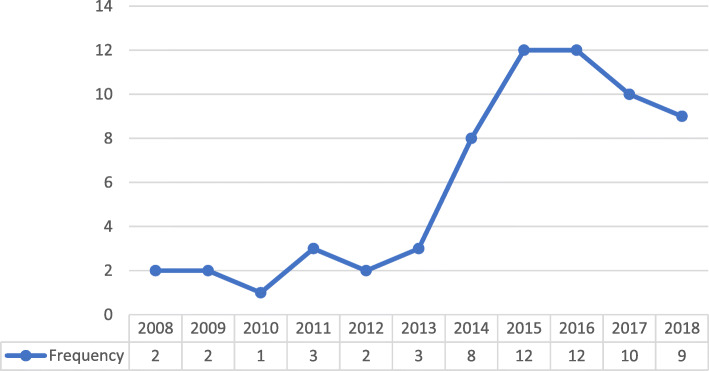
Frequency of publications by year

Most of the included studies were conducted in the USA [20, 26, 28, 32, 34, 41, 43, 51–53, 63, 66, 71, 73, 76–78, 82, 84–86], UK, [19, 27, 40, 47–50, 57, 61, 70, 74] and Canada [44, 54, 56, 60, 62, 64, 75, 87]. Primarily studies were conducted within a primary care, [8, 19, 20, 26–28, 32, 34, 35, 37, 38, 42, 43, 52, 53, 57–59, 62–64, 69, 76, 79, 85] or hospital setting [25, 31, 33, 39, 41, 45, 47, 49, 55, 56, 61, 65–67, 70, 72, 73, 75, 78, 80, 81, 84, 87] with others taking place across these settings [36, 48, 60, 82], at district level [50, 68, 74] or at a national level within health systems [40]. For some articles, it was unclear which care setting the research was conducted [44, 46, 51, 54, 71, 77, 83, 86]. Figure 2 illustrates the growing research interest in context within the field of implementation science, however, a slight decline is noted within the previous 2 years.

Thirty-four of the included studies used a qualitative approach [8, 19, 26, 31–33, 35–41, 43–45, 47, 49, 50, 52, 54, 56, 57, 59–64, 66–69, 71], 18 studies were purely quantitative [20, 25, 72–87], while 12 conducted a mixed methods evaluation [27, 28, 34, 42, 46, 48, 51, 53, 55, 58, 65, 70]. Studies using a qualitative approach commonly used interview methods, while quantitative studies predominantly employed surveys for data collection. Most engaged in primary data collection, however, four studies conducted a retrospective evaluation of existing data sources [19, 66, 69, 71]. Table 2 summarises the results of the quality appraisal using the appropriate sections of the MMAT for study design.

### Narrative synthesis

Given the heterogeneity of included papers, studies were categorised by the quality of the definition provided, the depth of application for the context assessment chosen and the unit of analysis employed and are described in the following narrative synthesis.

### Definition of context

Studies used a variety of definitions to describe context. Of the 64 articles, 60% did not meet our classification of providing a rich definition for this construct (*n* = 38). Instead much of the literature simply listed contextual factors [25, 37, 42, 48, 50, 56, 58, 61, 63, 64, 69, 73, 77, 78, 80, 81, 83, 85, 87] or sub-elements of a framework that includes context [26, 32, 41, 43, 45, 46, 49, 51–54, 59, 60, 66, 67, 72, 75, 79, 86]. Of the remaining articles, 11 provided a comprehensive definition of context generally [8, 19, 27, 32, 35, 38–40, 55, 68, 70, 71, 84], while 15 defined an aspect of context [20, 28, 31, 33, 36, 44, 47, 57, 65, 74, 76, 82, 84, 85, 87]. Definitions from the included papers are categorised in terms of their quality as outlined in Table 1. The full definitions of context included in each paper are presented in Table S1.

#### Listing contextual factors

Most of the available literature on context described the construct by merely recording associated factors. Some studies defined context by identifying a broad list of features such as the political or economic environment [48, 56, 69]. One paper associated context generally with multiple levels of the healthcare system including the *“team working on the project, the microsystem in which they function … the organisation in which they work, and the external environment”* [42](p717). Others provided specific examples of contextual conditions that relate to these domains and system levels. To effectively synthesise the studies included in this subsection, definitions will be categorised in accordance with individual, team, organisational and external characteristics.

##### Individual

Some studies highlight the perceptions and attitudes of individuals as key components of context. Individual autonomy [25], self-efficacy [81], individual knowledge, attitudes and beliefs [87] are described as influential contextual conditions within included papers. Additional factors identified include the interpretations of individuals about the initiative [50], with one study simply acknowledging the socioeconomic background of participants as a contextual determinant [37].

##### Team

Other studies adopt a different perspective suggesting that the perceptions of teams rather than individuals are important contextual features. Spitzer-Shohat et al. [58] propose that the team’s perceptions of the availability of resources, their capabilities in succeeding as well as the social relationships between team members and management are key contextual drivers. Team characteristics and teamwork [73] as well as team stability, morale, workload and staffing [61] were also identified as key contextual characteristics.

##### Organisational

Most of the literature incorporates organisational features within definitions of context. Whilst Chan et al. [73] and Chiu and Ku [81] adopt a broader perspective, referring to organisational support or facilitating factors, others specify this support in terms of organisational resources [25, 63, 64, 73, 78, 80, 83, 87]. While some studies broadly outline the availability of these resources [50, 64, 73, 83, 87], others specify this to include relevant *“expertise”* [63] and incorporate the ownership of resource allocation [78]. Others specify that organisational resources incorporate adequate training [25], staffing, time and space [80].

Leadership is listed as a key contextual feature for many studies included in this section of the review. However, Yamada et al. [87] is the only paper to classify this characteristic as an organisational factor. Similarly, four studies specify organisational norms as a contextual determinant [50, 63, 83, 85] with two acknowledging the workload and demands of organisations [50, 77]. Additional contextual factors cited include organisational climate [85], organisation size, change and structure [50], and organisational capacity [83].

##### External environment

Five studies include aspects of the external setting within their definition of context. Health policy [64], public reporting structures [61], the structure and dynamics of the wider health service [37, 80] and the capacity of the community [78] are outlined as influential contextual conditions. For some studies it is unclear whether the contextual driver mentioned was a micro, meso or macro level feature as these factors may be evident across multiple levels of the health system. Leadership was outlined as a contextual determinant in much of the literature included [61, 63, 64, 73, 80]. This concept is further described as the presence of “*champions”* during implementation [63], or the support of leaders [73] or management [25, 61]. Similarly, social relationships are cited within three definitions [33, 36, 69]. Additional multi-level contextual factors include culture, feedback mechanisms [80], support [83], learning climate [64], and compatibility [81] with two studies outlining contextual drivers specific to their study [25, 77].

##### Listing sub-elements of a framework that includes context

Most of the papers list elements of a framework that includes context. Seven of the articles define context by listing elements of the PARiHS framework; culture, leadership and evaluation [26, 41, 46, 52, 66, 72, 75]. Two of these studies, provide a brief description of these determinants [52, 66], with one outlining factors of a prevailing context [52]. In contrast, Arney et al. [32] refer to the PARiHS framework within their definition but do not list its sub-elements, rather they provide a broad perspective of context as the physical and social climate at each site. Similarly, Presseau et al. [54] define context as the physical location, however, refer to the Theoretical Domains Framework (TDF) within this description.

Three articles use the outer and inner setting domains of the CFIR to describe context [59, 60, 86]. While two of these studies focus on broad factors relating to context [60, 86], VanDevanter et al. [59] list contextual drivers specific to their study. Two papers utilise the Exploration, Preparation, Implementation, Sustainment (EPIS) model to define context. Like VanDevanter et al., Gadomski et al. [43] provides a tailored definition of the outer context that is specific to their study, while Padwa et al. [53] provides a description of outer and inner context that is broadly applicable.

Only one study used elements of the Model for Understanding Success in Quality (MUSIQ) framework within their description of context [49]. Within this definition the setting and environment are noted to be distinct features with leadership listed as an additional factor. Georgiou & Westbrook [45] is the only study included to use elements of the Kaplan’s 4C’s framework to describe context, where the setting and culture are acknowledged as key contextual drivers [45].

Two papers used aspects of the sociotechnical framework within their definition of context [51, 67]. Workflow and communication are factors listed within both studies, however, one includes costs and management as additional contextual conditions [67], while the other acknowledges internal and external policies, culture and monitoring systems as contextual drivers [51]. One paper [79] employed a bespoke framework developed from the theoretical approaches of Cretin et al. [91] and Lin et al. [92] which incorporates the organisational contextual factors of culture, commitment to quality improvement and climate.

##### Provides a meaningful definition of general context

Eleven studies provided a broad but rich definition of context. Within many of these definitions, the influence of context on implementation success is acknowledged as critical [19, 27, 35, 38–40, 55, 68, 70, 71]. Some papers elaborate that contextual factors are the barriers and facilitators encountered during implementation [39, 40, 70, 71]. Within the included papers context related to the pre-existing structural and organisational factors of the setting [27], or also included dynamic determinants that arose as implementation progressed [70]. Some studies suggest that contextual factors can be internal or external to the intervention [8, 39, 40, 70], while others propose that these conditions have no association with the initiative but rather with micro, meso and macro levels of the health system [19, 35, 38, 55, 68, 71]. Context is categorised by some papers as the internal (organisational level features) and external environment (macro level conditions) [38, 55] while other studies also incorporate individual characteristics within their definitions of context [35, 39]. Conversely, one study [8] adopts elements of a conceptual framework [93] to assist in defining context which encompasses the setting, the behavioural environment, the language and extra-situational context. Only three studies detail the possible interactions between these system levels within their definition of context [8, 35, 71].

##### Provides a meaningful definition of an element of context

Fifteen studies included in this review provide a rich definition of an aspect of context. Five studies provide a comprehensive definition of contextual characteristics [28, 34, 65, 76, 82] while the remaining papers offer a rich definition for sub-elements of a framework that includes context [20, 31, 33, 36, 44, 47, 57, 62, 74, 84].

All five studies that define an aspect of context conceptualise organisational climate and/or organisational culture as important constructs. Three studies employ both concepts and use similar definitions; the norms that characterise how work is done within the organisation (organisational culture) and the shared perceptions of how the work environment affects the wellbeing of staff (organisational climate) [28, 34, 76]. Whilst Ehrhart et al. [82] focuses exclusively on organisational climate, unlike other studies, it is specified to include molar (totality of the organisation) and focused climates (components of an organisation). In addition to defining organisational culture, Erasmus et al. [65] refers to organisational trust as an important contextual determinant.

Six papers use a rich definition of an element of the TDF to describe context [31, 33, 47, 57, 62, 74]. Five of these studies [31, 33, 47, 57, 62] use the same definition created by Cane et al. [94] to describe this concept; *“any circumstance of a person’s situation or environment that discourages or encourages the development of skills and abilities, independence, social competence, and adaptive behaviour”* (p14). One study adapted this definition to apply to their specific topic of study [74].

Other papers describe context by offering a generic definition for the construct while also detailing the context relevant aspects of the PARiHS framework [36, 44, 84]. Some studies introduce context as characteristics of the environment in which the proposed change will occur [36, 84], while another describes context as a multi-dimensional construct that can be referred to as *“anything that cannot be described as an intervention or its outcome”*, incorporating system level factors [44] (p2). Each study proceeds to detail culture, leadership and evaluation (sub-elements of the PARiHS framework). Bergstrom et al. [36] offers a detailed definition for each of these contextual drivers, while the remaining studies describe characteristics of receptive contexts; an organisational culture open to change, supportive leadership encouraging staff engagement and facilitates change and feedback mechanisms [44, 84].

Fernandez et al. [20] employed a pre-existing definition of the inner setting domain of CFIR to describe context. The inner setting is noted to incorporate innovation and organisational characteristics that can influence the likelihood of adoption, ultimately impacting implementation success.

Tables 4, 5, 6, 7 and 8 present the contextual factors included across the definitions of reviewed papers. For some articles it was not possible to isolate this information due to the broad definitions provided. However, for the remaining articles, contextual features are divided in terms of their relevance to micro, meso and macro levels of the healthcare system with some determinants recognised as applicable across multiple domains. Fourteen contextual factors relevant to individuals or teams were identified with individual perceptions for the implemented initiative the most cited determinant (Table 4 and 5). Seven organisational features were extracted with organisational culture the most commonly listed (Table 6). While five macro level factors were identified from the external environment, with political drivers such as policy the most commonly recorded feature (Table 7). The most common contextual determinants relevant to multiple levels of the health system were culture, leadership (Table 8) and resources (Tables 5, 6, 7 and 8).

**Table 4:** Individual contextual factors identified within the definitions of included papers

Article citation	Perceptions/ attitudes	Autonomy	Involvement	Socioeconomic background	Self-efficacy	Experience	Commitment
**Lists contextual factors**							
Abdekhoda et al. (2015) [25]	✓	✓	✓				
Bocoum et al. (2017) [37]				✓			
Chiu & Ku (2015) [81]	✓				✓		
Higgins et al. (2015) [50]	✓						
**Lists sub-elements of a framework that includes context**							
Gadomski et al. (2014) [43]	✓						
Padwa et al. (2016) [53]	✓						
Vandevanter et al. (2017) [59]	✓						
**Provides a meaningful definition of general context**							
Belaid & Ridde (2015) [35]	✓					✓	
Cheyne et al. (2013) [40]	✓				✓		
Rabbani et al. (2011) [55]	✓	✓					✓
**Total:**	**9**	**2**	**1**	**1**	**2**	**1**	**1**

**Table 5:** Team based contextual factors identified within the definitions of included papers

Article citation	Teamwork	Team resources	Team skills	Team relationships	Team stability	Team morale	Team workload
**Lists contextual factors**							
Chan et al. (2011) [73]	✓						
Spitzer-Shohat et al. (2018) [58]		✓	✓	✓			
Williams et al. (2016) [61]					✓	✓	✓
**Provides a meaningful definition of an element of context**							
Bergstrom et al. (2012) [36]	✓						
**Total**	**2**	**1**	**1**	**1**	**1**	**1**	**1**

**Table 6:** Organisational contextual factors identified within the definitions of included papers

Article citation	Organisational resources	Organisational culture	Organisational climate	Organisational support	Organisational characteristics	Organisational leadership	Organisational Trust
**Lists contextual factors**							
Almblad et al. (2018) [80]	✓						
Beidas et al. (2015) [85]		✓	✓				
Chan et al. (2011) [73]				✓			
Guerrero et al. (2015) [77]					✓		
Higgins et al. (2015) [50]	✓	✓			✓		
Hoffman & Rodriguez (2015) [78]	✓						
Huijg et al. (2014) [83]	✓	✓		✓			
Yamada et al. (2017) [87]	✓		✓			✓	
Yip et al. (2016) [63]		✓					
**Lists sub-elements of a framework that includes context**							
Arney et al. (2018) [32]			✓			✓	
Griffin et al. (2017) [49]	✓						
Iribarren et al. (2015) [67]				✓	✓		
Lemmens et al. (2009) [79]			✓	✓			
Menon et al. (2014) [51]		✓			✓		
Naik et al. (2015) [52]		✓					
Padwa et al. (2016) [53]					✓		
VanDevanter et al. (2017) [59]					✓		
**Provides a meaningful definition of an element of context**							
Beidas et al. (2014) [34]		✓	✓				
Ehrhart et al. (2014) [82]			✓				
Erasmus et al. (2017) [65]			✓				✓
Fernandez et al. (2018) [20]		✓			✓		
Gagliardi et al. (2014) [﻿56﻿]		✓					
Glisson et al. (2008) [76]		✓	✓				
Kramer et al. (2017) [28]		✓	✓				
**Provides a meaningful definition of general context**							
Belaid & Ridde (2015) [35]	✓						
Burau et al. (2018) [38]	✓				✓		
Hansen et al. (2011) [8]					✓		
Rabbani et al. (2011) [55]		✓			✓		
**Total:**	**8**	**12**	**9**	**4**	**10**	**2**	**1**

**Table 7:** External contextual factors identified within the definitions of included papers

Article citation	Economic environment	Health system characteristics	Political environment	Social environment	External incentives
**Lists contextual factors**					
Almblad et al. (2018) [80]	✓				
Bocoum et al. (2017) [37]		✓			
Durbin et al. (2016) [64]			✓		
Hoffman & Rodriguez (2015) [78]	✓				
Williams et al. (2016) [61]			✓		
**Lists sub-elements of a framework that includes context**					
Douglas (2016) [86]	✓		✓	✓	
Gadomski et al. (2014) [43]				✓	
Menon et al. (2014) [51]			✓		
Padwa et al. (2016) [53]	✓		✓	✓	
VanDevanter et al. (2017) [59]			✓		
Ware et al. (2018) [60]	✓		✓		✓
**Provides a meaningful definition of general context**					
Baron & Newman (2016) [70]	✓		✓	✓	
Burau et al. (2018) [38]	✓		✓	✓	
Rabbani et al. (2011) [55]	✓		✓	✓	
Vanderkruik & McPherson (2017) [71]			✓		
**Total**	**8**	**1**	**11**	**6**	**1**

**Table 8:** Multi-level contextual factors identified within the definitions of included papers

Article citation	Resources	Leadership	Management support	Culture	Evaluation	Social capital	Learning climate	Compatibility	Implementation setting
	**Lists contextual factors**								
Abdekhoda et al. (2015) [25]	✓		✓						
Almblad et al. (2018) [80]		✓		✓	✓	✓			
Chan et al. (2011) [73]	✓	✓							
Chiu & Ku (2015) [81]						✓			
Durbin et al. (2016) [64]	✓	✓					✓		
Williams et al. (2016) [61]		✓	✓						
Yip et al. (2016) [63]	✓	✓							
	**Lists sub-elements of a framework that includes context**								
Bokhour et al. (2015) [26]		✓		✓	✓				
Cummings et al. (2010) [75]		✓		✓	✓				✓
Douglas (2016) [86]				✓					
Drainoni et al. (2016) [41]		✓		✓	✓				
Förberg et al. (2016) [72]		✓		✓	✓				
Gadomski et al. (2014) [43]									✓
Georgiou & Westbrook (2009) [45]				✓					✓
Gibb (2013) [46]		✓		✓	✓				
Griffin et al. (2017) [49]	✓	✓		✓					✓
Hill et al. (2017) [66]		✓		✓	✓				
Iribarren et al. (2015) [67]	✓					✓			
Lemmens et al. (2009) [79]				✓					
Menon et al. (2014) [51]					✓				
Naik et al. (2015) [52]					✓	✓			
Padwa et al. (2016) [53]		✓			✓			✓	
Presseau et al. (2017) [54]									✓
VanDevanter et al. (2017) [59]								✓	
Ware et al. (2018) [60]						✓			
	**Provides a meaningful definition of an element of context**								
Beenstock et al. (2012) [74]	✓								
Bergstrom et al. (2012) [36]		✓		✓	✓				
Gagliardi et al. (2014) [﻿56﻿]		✓		✓					
Obrecht et al. (2014) [84]									✓
	**Provides a meaningful definition of general context**								
Belaid & Ridde (2015) [35]						✓			
Cheyne et al. (2013) [40]				✓					
Hansen et al. (2011) [8]						✓			✓
Rabbani et al. (2011) [55]	✓	✓		✓		✓			
**Total**	**8**	**16**	**2**	**15**	**11**	**8**	**1**	**2**	**7**

By synthesising the definitions included in this review, the findings generate a broad definition of context. Context is defined as a multi-dimensional construct encompassing micro, meso and macro level determinants that are pre-existing, dynamic and emergent throughout the implementation process. These factors are inextricably intertwined, incorporating multi-level concepts such as culture, leadership and the availability of resources.

### Application of context measures

**Fig. 3:**
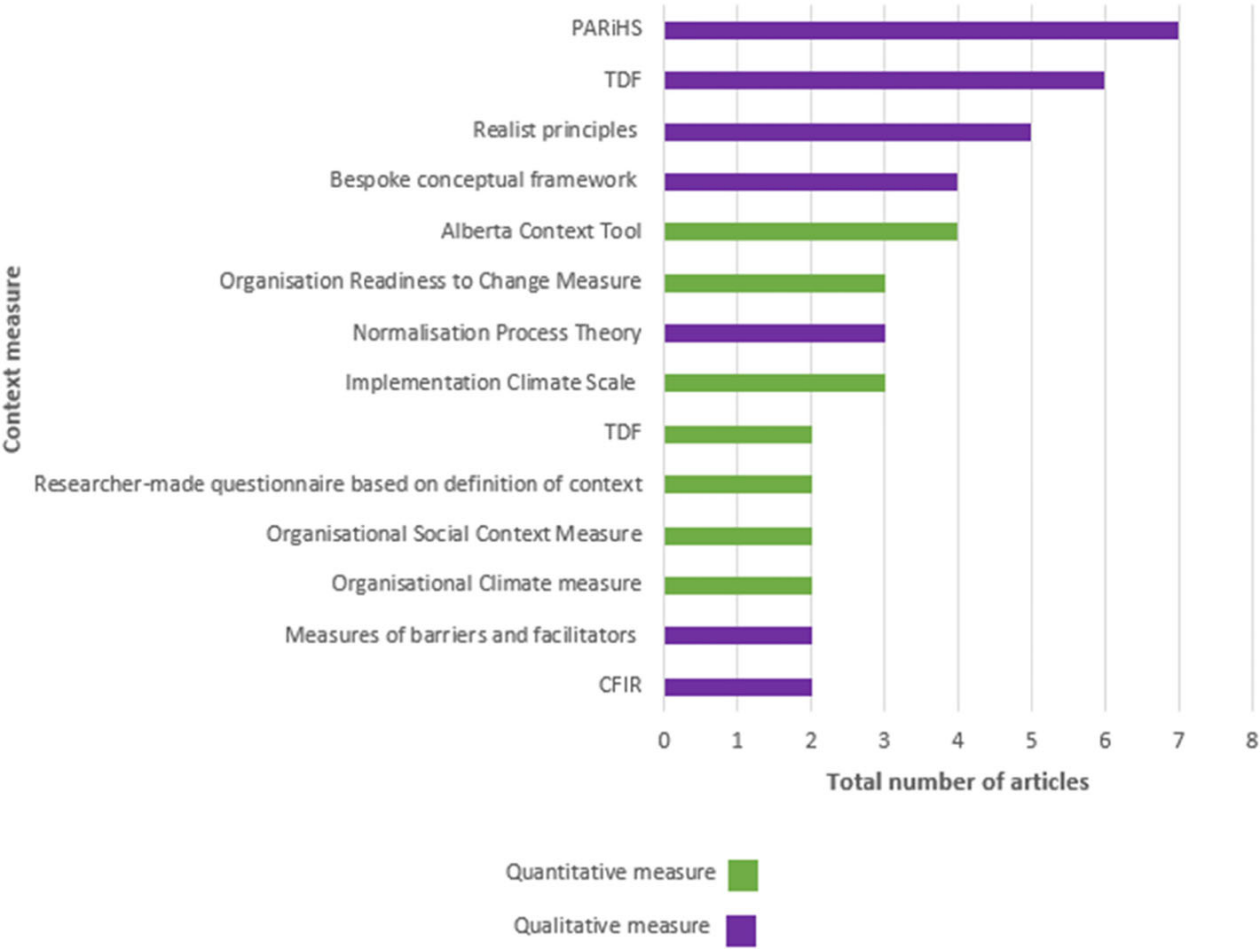
Categorisation and Frequency of Context Measures

Among the 64 articles included in this review, over 40 approaches to assess context were employed. Within quantitative papers, 22 context measures were identified with the Alberta Context Tool the most frequently applied (*n* = 4). Some measures were used in more than one study and are presented in Fig. 3. Most qualitative papers used frameworks to guide their context assessment with the PARiHS the most frequently applied (*n* = 7). Among the qualitative articles, 16 different approaches to assessment were used and the most highly cited are also represented in Fig. 3. Mixed methods studies used diverse approaches to assess context. Some authors used frameworks to inform the qualitative and quantitative methods chosen while others used quantitative tools to inform qualitative data collection.

#### Depth of measure application

The depth of application for all context assessment methods was appraised in relation to the application of the measure in the methods of each study and whether the measure was used to investigate an association between context and implementation success (Table 3). 50 of the included studies used their chosen context measure(s) to guide data collection, descriptive analysis and to investigate the association between context and implementation success. No study used measures to inform data collection alone while one study used their chosen assessment method for descriptive analysis only [27]. Whilst two studies used their context measures to guide data collection and descriptive analysis [31, 37], eleven used their assessment methods to inform data collection or data analysis and to investigate the association between context and implementation success [19, 26, 44, 48, 55, 56, 66, 70, 73, 78, 81].

### Unit of analysis

**Fig. 4:**
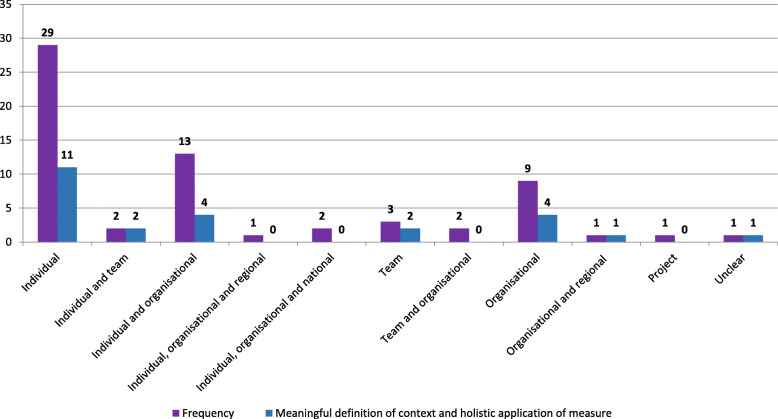
Unit of analysis

Despite the majority of included studies incorporating characteristics from multiple levels of the health system within their definition of context, the majority chose to analyse the construct at an individual level [25, 26, 31–33, 41, 43–47, 52, 54, 56, 57, 59, 60, 62, 68, 69, 72, 74, 76, 79, 76, 81, 83, 84, 86, 87] (Fig. 4). Some articles aggregated individual level findings to either a team [75, 82] or organisational level [8, 27, 28, 34, 36, 37, 48–51, 53, 67, 85] while three articles measured context at multiple levels of the healthcare system: individual, organisational and national [19, 63] or regional level [78]. Nine papers examined context exclusively at an organisational level [38–40, 55, 58, 61, 64, 65, 77], three assessed context using the team as the unit of analysis [20, 71, 80], while two paper analysed context at both a team and organisational level [42, 73]. The remaining three studies examined the construct at an organisational and regional level [35] or project level [66] with the unit of analysis unclear for one study [70]. Irrespective of the level of analysis, as outlined in Fig. 4, most studies failed to provide a comprehensive definition of context or apply their chosen measure holistically to all aspects of study design (data collection, descriptive data analysis and exploring the association between context and implementation success).

## Discussion

The objective of this systematic review was to explore how context is defined and measured within implementation science literature. The findings demonstrate the variability with which context is defined, assessed and analysed. Studies varied in terms of the level of detail and explanation offered within their definition of context with only 40% of included papers meeting our classification of providing a rich definition. Inconsistencies were also acknowledged in the approaches used in assessing context with over 40 methods identified within the 64 included papers. Therefore, it is not surprising that context remains poorly understood within implementation research [13].

Consistent with previous literature, this review found that most studies provided a narrow conceptualisation of context and simply listed contextual determinants associated with the construct [16–18]. Like Nilsen and Bernhardsson’s [18] scoping review, this systematic review examined the contextual factors cited within the definitions of included studies. There are some similarities between the contextual determinants identified and those previously reported [18]; social relations, leadership, organisational culture and climate. However, this review found additional features that were common across included studies; individual perceptions of the implementation effort, organisational characteristics, the wider political environment, and the multilevel concepts of culture and resources. This additional information may reflect the wider scope of this review (*n* = 64 papers included) as Nilsen and Bernhardsson restricted their search to literature conceptualising context within determinant frameworks (*n* = 22 publications included).

Few papers used team characteristics to define context [36, 58, 61, 73]. Unlike studies which listed determinants from an individual, organisation and/or external environment, only one contextual feature at a team level (teamwork) was shared across papers [36, 73]. This finding suggests that our understanding of team contextual determinants is limited as they have been almost entirely overlooked in the classification of context. This is surprising as teams are central to the organisational structure of healthcare, playing an integral role in care provision. Therefore, despite the findings suggesting an emphasis on organisational determinants, future developments within the field of implementation research requires the assessment of context at the team level.

Regardless of whether a study simply listed determinants related to context or provided a rich description, it is evident from the definitions included that context is a multifaceted term incorporating multiple levels of the healthcare system. Bergstrom et al. [36] argues that the influence of internal context on implementation success cannot be assessed without examining the impact of the wider health system in which these factors are situated. Literature suggests that the relationship between system components is of greater importance than the individual features themselves [95, 96]. This is particularly applicable when introducing an initiative within healthcare which is characterised by an infinite combination of care activities, events, interactions and outcomes [96–99]. Implementation researchers would benefit from employing a complexity science perspective when designing future studies. Complexity science recognises the interconnections of system components, acknowledging that the health system is made up of “*messy, fuzzy, unique and context embedded problems*” [100, 101] (p801). This perspective aligns with the study of context acknowledging that implementation is impacted by the configuration of local services and the variation in the attitudes and norms of those expected to adopt the envisioned change [102].

Although the findings of this review support the opinion that indistinct boundaries exist between system levels and implementation [9, 103, 104], intervention characteristics appear to be distinct features rarely encompassed within conceptualisations of context. Most of the studies identified that context incorporates determinants independent of the intervention with few mentioning the innovation itself. This finding is consistent with previous literature that views context as *“everything else that is not the intervention”* [18, 105] (p605).

By generating a common definition of context, the findings of this review have the potential to improve the consistency with which the term *‘context’* is used within implementation research. By using this broad definition as outlined in the results as a guideline, greater consistency will likely enable an enhanced understanding of the construct and direct attention to the multi-level nature of context. However, to ensure clarity, future research should expand this developed definition to incorporate the specific contextual factors they are measuring in their study, whilst also specifying the level (i.e. individual, team, organisation, system) to which these factors pertain.

There was heterogeneity observed in how context is assessed within implementation science literature. Although the papers included highlight the benefits in using context assessments, the lack of standardisation restricts our ability to compare the findings, impacting our understanding of the construct. Quantitative elements of included papers mostly used validated, context sensitive surveys to examine context or used contextual features outlined in their definition or a context relevant framework to inform survey development. Although the questionnaires used in these studies heightened the reach and possible generalisability of findings, studies using a qualitative approach enabled a greater exploration into the richness and complexity of the construct as relevant contextual determinants could emerge rather than those narrowly specified in some quantitative assessments. For qualitative aspects of included studies, context assessments were mostly used to guide data collection through the development of topic guides and/or employed deductively during data analysis to inform the development of coding templates.

Despite the variation in how context is measured within implementation science research, most studies applied their context assessment holistically to all aspects of study design (data collection and descriptive analysis) and investigated the association between context and implementation success. While the PARiHS was the most frequently employed framework within this review, qualitative studies that used the TDF to inform their assessment of context provided the most detailed description of the construct. A possible explanation for this might be the uniformity with which context was defined across papers and the consistency of the approach (framework informed data collection and analysis). Articles using this method recognised that it enabled a greater understanding of implementation determinants [47], improved the efficiency with which data could be coded [33], and promoted the rigour and trustworthiness of the research [57]. Development within the field would benefit from employing a similar approach in designing future studies to ensure a comprehensive evaluation of context is achieved.

This review illustrates inconsistencies in how context is defined and how it is subsequently analysed. Although only 10 articles listed individual contextual factors within their definition of context, 45% of all included papers analysed context exclusively at an individual level. Just four papers [50, 65, 73, 77] were consistent with their approach; explicitly listing contextual factors and the relevant system level in their definition and analysed their data accordingly. The remaining studies either used broad multi-level system components within their definition of context which could not be specified to a level of analysis or included system-level contextual factors within their definition which were not analysed at the appropriate level. For example, Yamada et al. [87] exclusively lists organisational factors within their definition of context but employ an individual level of analysis. However, of the four papers that defined and analysed context consistently, Erasmus et al.’s [65] study was the only paper to also provide a rich definition of context, use their chosen context assessment holistically and analyse the data appropriately. Future research must ensure consistency with how context is defined, measured and analysed within the field of implementation science. Greater clarity will enhance the rigour associated with studies exploring context, developing the fields *‘true’* understanding of how context influences implementation.

### Limitations

Although this study provides a broader conceptualisation of context in comparison to previous literature, the findings are limited to definitions retrieved through the search strategy applied. One challenge is the multitude of terms used to describe *‘implementation science’*. Despite the final search yielding thousands of articles, the endeavour to strike a balance between a sensitive and specific search strategy increases the possibility that relevant articles may have been omitted from this review. The inclusion of purely empirical studies heightens the risk of publication bias as the grey literature was not appraised. However, we hope to have limited the impact of these challenges by using previous literature to inform the search strategy and scanning reference lists of included articles to retrieve additional relevant studies. It is hoped that this approach has ensured that a comprehensive synthesis of the best available evidence has been presented.

## Conclusion

This review set out to systematically investigate how context is defined, measured and analysed within implementation science literature. The review confirms that context is generally not comprehensively defined and adds to the extant literature [16–18] by developing an operational definition to improve the consistency with which the term is used. Due to the variability in how context is assessed, it is recommended that a standardised approach using qualitative methods informed by a comprehensive framework is the most suitable assessment to explore the complexity of this phenomenon. Additionally, the need for researchers to define, assess and analyse context coherently was highlighted as most studies failed to use a consistent approach. Enhanced clarity and consistency when studying context, may result in improvements in implementation processes. A heightened understanding will help researchers appropriately account for context in research, enhancing the rigour and learning acquired which can aid in the translation of evidence-based healthcare interventions into routine practice.

## Supplementary information

**Additional file 1.** Search Strategy.

**Additional file 2.** Data extraction template.

**Additional file 3: Table S1.** Summary of included papers.

## Data Availability

Data analysed in this study is available through the journal articles cited herein.

## Abbreviations

PARiHS: Promoting Action on Research Implementation in Health Services
CFIR: Consolidated Framework for Implementation Research
EPIS: Evaluation, Planning, Implementation, Sustainability
PRISMA: Preferred Reporting Items of Systematic Reviews and Meta-Analyses
PROSPERO: database for protocol details for systematic reviews relevant to health and social care, welfare, public health, education, crime, justice, and international development, where there is a health-related outcome
MMAT: Mixed Methods Appraisal Tool
USA: United States of America
UK: United Kingdom
MUSIQ: Model for Understanding Success in Quality
TDF: Theoretical Domains Framework
