# Supplementary material for: Defining and assessing context in healthcare implementation studies: a systematic review
Source: BMC Health Serv Res. 2020 Jun 29;20:591. doi: 10.1186/s12913-020-05212-7 (PMC7322847; doi:10.1186/s12913-020-05212-7)
Supplement: Supplementary file 1 — Additional file 1. Search Strategy. [file 12913_2020_5212_MOESM1_ESM.docx]

**Additional File 1: Search Strategy**

| Databases | A |  | B |  | C |  | D |  | E |  | F |  | G | Combined search results |
| --- | --- | --- | --- | --- | --- | --- | --- | --- | --- | --- | --- | --- | --- | --- |
|  | Health* | **AND** | Context | **AND** | Contextual* | **OR** | Environment* | **AND** | Context NOT “in the context of” | **AND** | Measure* | **AND** | Implement* |  |
|  |  |  |  |  |  |  | OR |  |  |  | OR |  | OR |  |
|  |  |  |  |  |  |  | Setting* |  |  |  | Assess* |  | Knowledge translation |  |
|  |  |  |  |  |  |  |  |  |  |  | OR |  | OR |  |
|  |  |  |  |  |  |  |  |  |  |  | Evaluation* |  | Translational research |  |
|  |  |  |  |  |  |  |  |  |  |  |  |  | OR |  |
|  |  |  |  |  |  |  |  |  |  |  |  |  | Quality improvement |  |
|  |  |  |  |  |  |  |  |  |  |  |  |  | OR |  |
|  |  |  |  |  |  |  |  |  |  |  |  |  | Improvement science |  |
|  |  |  |  |  |  |  |  |  |  |  |  |  | OR |  |
|  |  |  |  |  |  |  |  |  |  |  |  |  | Diffusion of innovation* |  |
|  |  |  |  |  |  |  |  |  |  |  |  |  | OR |  |
|  |  |  |  |  |  |  |  |  |  |  |  |  | Organi#ation change |  |
|  |  |  |  |  |  |  |  |  |  |  |  |  | OR |  |
|  |  |  |  |  |  |  |  |  |  |  |  |  | Organi#ational change |  |
| **Medline** | 2285963 |  | 339458 |  | 30683 |  | 1292809 |  | 170527 |  | 5391335 |  | 419258 | 965 |
| **EMBASE** | 3,087,179 |  | 418,902 |  | 35,247 |  | 1,637,349 |  | 262,328 |  | 7,244,833 |  | 556,781 | 1558 |
| **CINAHL** | 902,507 |  | 102,803 |  | 13,727 |  | 318,416 |  | 10,836 |  | 1,058,202 |  | 160,427 | 125 |
| **PsycINFO** | 631,940 |  | 317,385 |  | 50,686 |  | 472,425 |  | 255,127 |  | 1,284,608 |  | 182,115 | 373 |
|  |  |  | | | | | | | | | | | **Total** | 3021 |

**Summary: “A” AND “B” AND “{C OR D}” AND “E” AND “F” AND “G”**
