# Supplementary material for: Defining and assessing context in healthcare implementation studies: a systematic review
Source: BMC Health Serv Res. 2020 Jun 29;20:591. doi: 10.1186/s12913-020-05212-7 (PMC7322847; doi:10.1186/s12913-020-05212-7)
Supplement: Supplementary file 2 — Additional file 2. Data extraction template. [file 12913_2020_5212_MOESM2_ESM.docx]

| **Study ID:** | |
| --- | --- |
| **Name of Reviewer** | |
| **Date of Data Extraction:** | |
| **Study Citation:** | |
| **Study Aim / Objective:** | |
| **Country of origin** | |
| **Study Setting:** | |
| **Study Design:** | |
| **Implementation focus:** | |
| **Definition of Context** | **Quality of Definition: (listing factors vs providing an operational definition)** |
| **Measure(s) of Context** | **Depth of Measure Application:**   - Measure used to guide data collection, analysis, both: - Investigation of outcomes (association between measure and implementation success): |
| **Unit of Analysis**  Individual  Team  Organisation  National system  International system | |

**Additional File 2: Data extraction template**
