# Supplementary material for: Defining and assessing context in healthcare implementation studies: a systematic review
Source: BMC Health Serv Res. 2020 Jun 29;20:591. doi: 10.1186/s12913-020-05212-7 (PMC7322847; doi:10.1186/s12913-020-05212-7)
Supplement: Supplementary file 3 — Additional file 3: Table S1. Summary of included papers. [file 12913_2020_5212_MOESM3_ESM.docx]

**Table S1. Summary of included papers**

| **Author, Year, Country** | **Aim** | **Setting** | **Study design** | **Implementation focus** | **Definition of context** | **Measure of context** |
| --- | --- | --- | --- | --- | --- | --- |
| Abdekhoda et al. (2015)  Iran | To determine the organisational contextual factors that may affect physicians’ acceptance of EMR^[[1]](#footnote-1)^ adoption. | Hospital | Quantitative (conceptual path model (SEM)) | EMR^1^ | Management support, adequate training, physician involvement, physician autonomy and doctor-patient relationship. | A questionnaire consisting of 30 structured questions on the elements outlined in the definition. |
| Almblad et al. (2018)  Sweden | To describe data of PEWS^[[2]](#footnote-2)^ registrations and to evaluate the implementation of PEWS^2^ by examining  adherence to clinical guidelines based on measured PEWS^2^, and to relate findings to work context. | Hospital- elective care, oncology and emergency departments | Quantitative (cross-sectional survey and data audit) | PEWS^2^ guideline | Context is defined in relation to sub-elements of the ACT^[[3]](#footnote-3)^; leadership, culture, evaluation, social capital, formal and informal interactions, structural and electronic resources and organisational  resources of staffing, time and space. | ACT^3^ |
| Al Shemeili et al. (2016)  Abu Dhabi | To describe and understand health professionals’ views and experiences of medicines management  healthcare structures, processes and outcomes for elderly, hospitalised patients. | Hospital | Qualitative (interviews) | Medicines management protocols | Circumstances of a person’s situation or environment that discourages or encourages the development of skills and abilities, independence, social competence, and adaptive behaviour.  (Definition based on the TDF^4^) | TDF^[[4]](#footnote-4)^ informed interview guide and analysis of data |

| **Author, Year, Country** | **Aim** | **Setting** | **Study design** | **Implementation focus** | **Definition of context** | **Measure of context** |
| --- | --- | --- | --- | --- | --- | --- |
| Arney et al. (2018)  USA | To conduct a formative evaluation to inform the implementation of the EPIC^[[5]](#footnote-5)^ intervention. | Primary care clinics | Qualitative (interviews) | EPIC^5^ an evidence-based interdisciplinary group medical appointment intervention to improve collaborative goal-setting in patients with treated but uncontrolled diabetes. | Physical and social climate at each site; how EPIC^5^ can be best embedded into routine care. | Interview questions aligned to PARiHS^[[6]](#footnote-6)^ framework. |
| Bain et al. (2015)  Australia | To assess health professionals’ self-reported use and knowledge of bi-national guidelines for antenatal magnesium sulphate for foetal neuroprotection over time, and to explore local barriers and enablers to implementation using theoretical domains related to behaviour change. | Women’s and Children’s Hospital | Qualitative (interviews) | Bi-national guidelines for antenatal magnesium sulphate for foetal neuroprotection. | Any circumstance of a person’s situation or environment that discourages or encourages the development of skills and abilities, independence, social competence, and adaptive behaviour.  (Definition based on the TDF^4^) | TDF^4^ informed the interview guide development and analysis |

| **Author, Year, Country** | **Aim** | **Setting** | **Study design** | **Implementation focus** | **Definition of context** | **Measure of context** |
| --- | --- | --- | --- | --- | --- | --- |
| Baron & Newman (2016)  UK | To report a process evaluation for a mobile telehealth RCT^[[7]](#footnote-7)^ focusing on recruitment, implementation,  fidelity, and context, as well as the relationships between these domains. | Hospital-diabetes clinic | Mixed methods | Mobile telehealth intervention | Aspects of the larger social, political, and economic environment that may influence the study.  Contextual factors are barriers or facilitators to the study that exist prior to, or that emerge during, implementation. They can be internal or external to the intervention, and they can interact with an intervention in complex ways, impacting generalisability and moderating study outcomes. | Data on contextual factors affecting recruitment, intervention delivery, and fidelity were taken from meetings or researchers’ field notes, as well as from e-mail communications among the research team |
| Beenstock et al. (2012)  UK | 1) To investigate the perceived implementation difficulties of midwives, working in different roles and locations, in providing smoking cessation advice to pregnant women who smoke  2) To explore the relationship between the self-reported behaviour of referring women to smoking-cessation services and demographic  and professional variables. | Hospital trusts in North East England | Quantitative (cross-sectional survey) | NICE guidelines on smoking cessation for pregnant women | Environmental context and resources: Are resources available for midwives to support pregnant women who smoke to stop? To what extent do resources help or hinder supporting pregnant women who smoke to stop?  (Definition based on the TDF^4^). | Questionnaire was designed to assess the domains of the TDF^4^ |

| **Author, Year, Country** | **Aim** | **Setting** | **Study design** | **Implementation focus** | **Definition of context** | **Measure of context** |
| --- | --- | --- | --- | --- | --- | --- |
| Beidas et al. (2014)  USA | To demonstrate the use of mixed methods to deepen our understanding of organsational social context. | Community (child-serving agencies) | Mixed methods | The implementation of EBP^[[8]](#footnote-8)^: the use of cognitive-behavioural, family, and psychodynamic techniques | Organisational culture; refers to shared employee perceptions around “the behavioural expectations and norms that characterise the way work is done in an organisation”.  Organisational climate refers to shared employee perceptions around “the psychological impact of their work environment on their own personal wellbeing”. | Observational data - field notes included the physical atmosphere (e.g., temperature, building appearance), the professional atmosphere (e.g., collegiality among staff), and general impressions about the visit as these were a priori constructs of interest.  OSC^[[9]](#footnote-9)^ quantitative measure also used |

| **Author, Year, Country** | **Aim** | **Setting** | **Study design** | **Implementation focus** | **Definition of context** | **Measure of context** |
| --- | --- | --- | --- | --- | --- | --- |
| Beidas et al. (2015)  USA | To explore the relative contribution of clinician and organisational factors on therapist self-reported use of cognitive-behavioural, family, and psychodynamic techniques within a large-scale effort to increase use of EBP^8^s in an urban public mental health system serving youth and families. | Community mental health agencies | Quantitative (cross-sectional survey) | Adoption of cognitive behavioural, family and psychodynamic techniques | Organisational culture (i.e., shared employee perceptions around expectations and norms) and organisational climate (i.e. psychological impact of the work environment on individual well-being). | OSC^9^ measure  ICS^[[10]](#footnote-10)^ measure  ILS^[[11]](#footnote-11)^ |
| Belaid & Ridde  (2015)  Burkina Faso | To evaluate the disparities observed in the coverage of facility-based deliveries after implementation of the EmONC^[[12]](#footnote-12)^ policy. | Primary healthcare centres | Qualitative (multiple case study-interviews, observations, focus groups) | National policy: EmONC^12^ policy | All elements at the microlevel that might have an influence on the use of skilled birth attendance. These factors can be found at the individual level, such as perceived quality of care (satisfaction, staff behaviour) and experience of childbirth in health facilities, and at the  organisational level (health centres, human resources) and in the interactions between these two levels (relations between patients and health workers). | Conceptual framework based on 4 factors: sociocultural factors (marital status, ethnicity), perceived needs and benefits (health  knowledge, quality of care, information, prenatal care), economic accessibility (capacity to cope with costs associated with maternal  healthcare) and physical accessibility (transport, distance). These four determinants guided data collection, case descriptions and analysis. |

| **Author, Year, Country** | **Aim** | **Setting** | **Study design** | **Implementation focus** | **Definition of context** | **Measure of context** |
| --- | --- | --- | --- | --- | --- | --- |
| Bergström et al. (2012)  Uganda | To examine the perceived relevance of the sub-elements of the organisational context cornerstone of the PARIHS^6^ framework, and whether additional factors in the organisational context were perceived to  influence knowledge translation in Uganda. | Hospital and community health centres | Qualitative (focus group discussions and interviews) | Knowledge translation among midwives and managers | The environment or setting in which the proposed change is to be implemented and includes four sub-elements: receptive context, culture, leadership, and evaluation.  Culture is described as the degrees of clarity in values and beliefs, the level of regard for individuals, the organisational ‘drive’ (task versus learning), the degree of consistency in valuing relationships, teamwork, power, and authority, and the extent of recognition or reward that is provided or, simply put ‘the way we do things’.  Leadership summarises the nature of human relationships in the practice context.  Evaluation is defined as efforts aimed at determining as systematically and objectively as possible, the effectiveness and impact of health-related (and other) activities in relation to objectives and taking into account the resources and facilities that have been deployed in the activities being evaluated. | The PARiHS^6^ framework guided data collection and analysis |

| **Author, Year, Country** | **Aim** | **Setting** | **Study design** | **Implementation focus** | **Definition of context** | **Measure of context** |
| --- | --- | --- | --- | --- | --- | --- |
| Bocoum et al. (2017)  Burkina Faso | To document operational implementation of a pilot integration of a rapid test for syphilis screening into routine antenatal services and analyse the experience in order to draw lessons for scaling up maternal syphilis screening in Burkina Faso. | Primary health care facilities | Qualitative (interviews and observations) | Rapid test for anti-natal syphilis screening | Socioeconomic background, health system structure and dynamics, and other factors. | NPT^[[13]](#footnote-13)^ guided data collection and analysis |
| Bokhour et al. (2015)  USA | To understand barriers and facilitators to implementation of HIV testing and examine how different contextual factors and perception of  evidence relate to the relative success of the implementation effort. | Primary care clinics | Qualitative | HIV testing intervention | Context includes aspects of the culture leadership and evaluation at the site. | PARiHS^6^ framework guided data analysis.  Each site was also rated based on the extent to which the interviews revealed a high, medium, or low context for testing. |

| **Author, Year, Country** | **Aim** | **Setting** | **Study design** | **Implementation focus** | **Definition of context** | **Measure of context** |
| --- | --- | --- | --- | --- | --- | --- |
| Bradley & Griffin  (2016)  UK | To report the quantitative outcomes from the Well Organised Working Environment module, to identify the contexts, mechanisms and outcomes evident during the implementation to explain the varying experiences of staff as indicated by the quantitative analysis, and to explore the implications of  these findings for multiple stakeholders. | Community healthcare organisations | Mixed methods | Examines the implementation of the first module of the Productive Community Services programme called ‘The Well Organised Working  Environment’. | The pre-existing conditions and relationships in the organisational system that partner with the programme’s  mechanisms to make success or failure of the intervention more or less likely. | Realist evaluation principals used to guide analysis. |

| **Author, Year, Country** | **Aim** | **Setting** | **Study design** | **Implementation focus** | **Definition of context** | **Measure of context** |
| --- | --- | --- | --- | --- | --- | --- |
| Burau et al. (2018)  Denmark | To analyse the implementation of a structural health promotion intervention in community mental health organisations in different contexts in Denmark. | Community mental health | Qualitative (multiple case study design- interviews and document analysis) | Health promotion intervention called SLIPS^[[14]](#footnote-14)^ which aimed to improve the health of users through activities that were embedded in and facilitated by changes in the structure of provider organisations including strategies to service delivery at multiple levels and the physical environment. | All factors that affect processes of organisational change but are not part of the intervention itself.  External contexts: the political-administrative structures of community mental health services, economic resources and inter-organisational relations.  Internal contexts: the formal characteristics of provider organisation, including formal structure and size, previous experience with health promotion, staff and other economic resources. | Data on external and internal contexts came from literature on community mental health services, research reports, policy documents and statistics, documents specifically produced as part of the intervention, and supplementary interviews with the municipal/regional managers of community mental health services.  Conceptual Framework informed interview guide, NPT^13^ guided analysis |
| Busetto et al. (2017)  Germany | 1) To describe the implementation of an integrated geriatric care intervention at a German geriatric hospital.  2) To explore whether the application of a CMO^[[15]](#footnote-15)^based model provides insights into when and why beneficial outcomes can be achieved. | Hospital-geriatric | Qualitative (interviews) | Integrated care intervention | Context is understood as the setting in which the mechanisms are brought into practice and described by the barriers and facilitators encountered in the implementation process. Barriers and facilitators included; innovation, individual professional, patient, social context, organisational context, health system context and economic, political and legal context. | Realist evaluation principles-CMO^15^ model used as a framework for data collection, analysis and interpretation |

| **Author, Year, Country** | **Aim** | **Setting** | **Study design** | **Implementation focus** | **Definition of context** | **Measure of context** |
| --- | --- | --- | --- | --- | --- | --- |
| Chan et al. (2011)  USA | To demonstrate that a short instrument, the TCT^[[16]](#footnote-16)^, can provide reliable and valid contextual data for monitoring team progress within a quality improvement intervention. | Hospital-intensive care units | Quantitative (secondary cross-sectional and longitudinal analysis of data from a clustered RCT^7^) | Comprehensive Unit-based Safety Programme | Team characteristics, organisational support, teamwork, leadership support and available resources. | TCT^16^  PES-NWI^[[17]](#footnote-17)^  TFS^[[18]](#footnote-18)^ |
| Cheyne et al. (2013)  UK | To explore and explain the ways in which the KCND^[[19]](#footnote-19)^ programme worked or did not work in different maternity care settings. | National healthcare context-Health Boards with national stakeholders and consultant midwives | Qualitative (case study using multiple qualitative methods-interviews, focus group discussions and case report audit) | KCND^19^ programme-a maternity care programme that aimed to support normal birth by implementing multi-professional care pathways and making midwife led  care for healthy pregnant women the national norm. | The context may facilitate or impede the programme because it influences what people do and how they will act. Context within this study includes views about the KCND^19^ initiative, programme implementation and facilitation, current practice and culture, and enabling and constraining factors. | Topic guides informed by a realist framework (CMO^15^)  Realist principles guided data analysis |

| **Author, Year, Country** | **Aim** | **Setting** | **Study design** | **Implementation focus** | **Definition of context** | **Measure of context** |
| --- | --- | --- | --- | --- | --- | --- |
| Chiu & Ku (2015)  China | To study the role of voluntariness on the actual use of EHR^[[20]](#footnote-20)^. | Hospital | Quantitative (Cross-sectional survey) | A module;AHPN^[[21]](#footnote-21)^ | Context is defined in 3 sub-elements;  (1) individual context (computer anxiety, computer self-efficacy, computer attitude), (2) technological context (perceived usefulness and  perceived ease of use), and (3) implementation context (social influence, compatibility, and organisational facilitating conditions). | Survey included questions that measured the levels of voluntariness, technology acceptance and use, and attitude towards technology. |
| Cummings et al. (2010)  Canada | To report findings from a pilot study assessing the ACT^3^, an instrument designed to measure modifiable dimensions of organisational context and self-reported research utilisation. | Hospital-paediatric units | Quantitative (Cross-sectional survey) | Study of research utilisation | Context is understood to be the environment or setting in which the proposed change is to be implemented. Context is proposed to have three dimensions (culture, leadership and evaluation).  (Definition based on PARiHS^6^) | ACT^3^ |
| Douglas (2016)  USA | To apply the CFIR^[[22]](#footnote-22)^ to a measurement of current practice patterns of speech and language pathologists working in the skilled nursing facility environment in the state of Michigan. | Skilled nursing facility | Quantitative (descriptive and nonparametric correlational analyses) | Evidence-based speech, voice, swallowing, language, and cognitive-communicative treatments | The outer setting specifically refers to the economic, political and social context of an organisation and the inner setting refers to the structural, political and cultural contexts where intervention implementation will actually occur.  (Definition based on CFIR^22^) | Survey questions mapped to CFIR^22^ constructs  ORCA^[[23]](#footnote-23)^ |

| **Author, Year, Country** | **Aim** | **Setting** | **Study design** | **Implementation focus** | **Definition of context** | **Measure of context** |
| --- | --- | --- | --- | --- | --- | --- |
| Drainoni et al. (2016)  USA | 1)To understand the acceptance and uptake of the policy for nasal naloxone distribution  2)To identify facilitators and barriers of  success. | Hospital- ED | Qualitative (interviews) | Policy-nasal naloxone distribution | Context includes culture, leadership and evaluation  (definition based on PARiHS^6^ framework) | Used PARIHS^6^ to inform interview guide and guide data analysis |
| Durbin et al. (2016)  Canada | To describe the practice changes made to implement the health check, and to evaluate how the practice context affected the  implementation decisions. | Primary care | Qualitative (interviews and focus group) | Health check for adults with intellectual and development disabilities. | Factors internal and external to an organisation that influence implementation such as leadership, resources, learning climate, health policy. | Implementation log completed using questions and prompts based on evidence about implementation barriers  and facilitators.  Focus groups were conducted, and topics included barriers and facilitators encountered during implementation.  Analysis focused on assessing the influence of context on implementation decisions. |

| **Author, Year, Country** | **Aim** | **Setting** | **Study design** | **Implementation focus** | **Definition of context** | **Measure of context** |
| --- | --- | --- | --- | --- | --- | --- |
| Eboreime et al. (2018)  Nigeria | To explore the role of actors and context in the implementation and sustainability of DIVA QI project by comparing experiences between Nigerian local government areas (LGAs)  in Kaduna state. | Primary care | Mixed methods | DIVA QI model (diagnose-intervene-verify-adjust) | Contextual factors broadly relate to the team working on the project, the microsystem in which they function, local QI support and capacity, the organisation in which they work, and the external environment. | MUSIQ^[[24]](#footnote-24)^ guided data collection and qualitative data analysis |
| Ehrhart et al. (2014)  USA | To develop and test a measure of EBP^8^ implementation climate that would both capture a broad range of issues important for effective EBP^8^ implementation and be of practical use to researchers and managers seeking to understand and improve the implementation of EBPs^8^. | Mental health agencies | Quantitative (scale development EFA, CFA) | EBP^8^ implementation | Organisational climate has been defined as the shared meaning organisational members attach to the events, policies, practices, and procedures they experience and the behaviours they see being rewarded, supported, and expected.  Molar climates attempt to capture the totality of the organisational environment. Focused climate addresses the components of the organisational environment that are most relevant to achieve a specific outcome (such as internal organisational processes). | ICM^10^  Service climate was measured with eight items from Schneider and colleagues  OCM^[[25]](#footnote-25)^  Perceived Organisational Change measure  ORCA^23^ subscale |

| **Author, Year, Country** | **Aim** | **Setting** | **Study design** | **Implementation focus** | **Definition of context** | **Measure of context** |
| --- | --- | --- | --- | --- | --- | --- |
| Erasmus et al. (2017)  South Africa | To investigate the experience of policy implementation and, more specifically, the influence of street-level bureaucrats, organisational culture and organisational trust over the implementation of the South African UPFS^[[26]](#footnote-26)^ and PRC^[[27]](#footnote-27)^ policies. | Hospital | Mixed methods | UPFS^26^ and PRC^27^ policies | Organisational culture was understood as artefacts, values and assumptions that are to some extent shared by members of an organisation and that influence organisational functioning; and organisational trust refers to trust between different people and parts of an organisation, in this case staff and managers | Organisational trust inventory  Organisational culture survey-based on the competing values framework  For qualitative data researchers generated initial analysis codes from research objectives and relevant concepts (organisational culture, organisational trust and discretionary power) |
| Fernandez et al. (2018)  USA | To develop and test measures of constructs of the Inner Setting domain of the CFIR^22^. | Community clinics | Quantitative (survey development CFA) | Dissemination and implementation of evidence- based approaches for cancer prevention and control | Organisational (inner) context which included both antecedents for innovation and readiness for innovation. Some characteristics of organisations (e.g., structure, culture) influence the likelihood that an innovation will be successfully adopted and incorporated into their usual practice. Inner Setting can be described as the clinic or organisational context in which the intervention will exist  (Definition based on CFIR^22^ construct) | Practice Adaptive Resource Scale  ORCA^23^  OCM^25^  Community Clinical Oncology Program survey  Implementation Climate Assessment |

| **Author, Year, Country** | **Aim** | **Setting** | **Study design** | **Implementation focus** | **Definition of context** | **Measure of context** |
| --- | --- | --- | --- | --- | --- | --- |
| Förberg et al. (2016)  Sweden | To investigate the effects of implementing a clinical practice guideline for PVCs^[[28]](#footnote-28)^ in paediatric care in the format of reminders, integrated in the EPR^[[29]](#footnote-29)^, on PVC^28^-related complications, and on Registered Nurses’ self-reported adherence to the guidelines.  An additional aim was to study the relationship between contextual factors  and the outcomes of the intervention. | Paediatric hospital | Quantitative (cluster randomised design) | Clinical practice guideline for PVCs^28^ and computer reminder intervention | Context comprises three dimensions: culture, leadership, and evaluation  (Definition based on PARiHS^6^) | ACT^3^ |

| **Author, Year, Country** | **Aim** | **Setting** | **Study design** | **Implementation focus** | **Definition of context** | **Measure of context** |
| --- | --- | --- | --- | --- | --- | --- |
| Gadomski et al. (2014)  USA | To understand what motivated PCP’s^[[30]](#footnote-30)^  participation, what components of Project TEACH^[[31]](#footnote-31)^ led to changes in practice, what other factors contributed to implementation of these changes and what was the perceived impact on clinical outcomes. The primary goal was to provide insight into how to most effectively  implement this strategy of integrating mental health and primary care. | Primary care | Qualitative (interviews) | Project TEACH^31^, 2 programs to promote primary care  provider-mental health integration | Outer context: includes the service environment  (reimbursement for MH^[[32]](#footnote-32)^, psychotropic prescription formularies), inter-organisational relationships (availability of MH^32^ providers, ability to refer), consumer support (MH^32^ stigma or awareness).  Inner context: applies to the PCP’s^30^ motivation to participate, attitude toward MH^32^ treatment and intra-organisational factors (appointment times, office practice design and patient population served).  (Definition based on the EPIS^[[33]](#footnote-33)^ model) | EPIS^33^ model guided data collection and data analysis |

| **Author, Year, Country** | **Aim** | **Setting** | **Study design** | **Implementation focus** | **Definition of context** | **Measure of context** |
| --- | --- | --- | --- | --- | --- | --- |
| Gagliardi et al. (2014)  Canada | To identify contextual factors influencing IKT^[[34]](#footnote-34)^ in different healthcare planning or improvement programs and, based on the findings, generate a conceptual framework by which others could plan, promote, strengthen or evaluate IKT^34^. | Health service programs for colon cancer screening, prostate cancer diagnosis, and the treatment of pancreatic cancer | Qualitative (document analysis, observations, interviews) | Knowledge translation | Anything that cannot be described as an intervention or its outcome.  Context is comprised of an organisational culture  that is receptive to change, leadership that supports the involvement of individual staff, and evaluation and feedback mechanisms.  (Definition based on PARiHS^6^) | PARIHS^6^ sub-elements used to analyse data. |
| Georgiou & Westbrook (2009)  Australia | To investigate the effects of CPOE^[[35]](#footnote-35)^ on ED^[[36]](#footnote-36)^ work, communication practices and patient care using  Kaplan’s 4Cs as an orienting framework. | Hospital- ED^36^ | Qualitative (interviews) | A CPOE^35^ intervention | The background, setting and culture that impacts on system functioning.  (Definition was based on Kaplans 4Cs framework- communication, care, control, context) | Kaplan's 4C's evaluation framework guided interviews, observations and the analysis  **.** |

| **Author, Year, Country** | **Aim** | **Setting** | **Study design** | **Implementation focus** | **Definition of context** | **Measure of context** |
| --- | --- | --- | --- | --- | --- | --- |
| Gibb (2013)  Australia | 1) To describe characteristics of the workplace that were strengths or enablers of cultural and practice change and which would potentially enhance the team's ability to implement aspects of TeamSTEPPS and, furthermore, sustain the practice change.  2) To identify cultural obstacles (habitual behaviours and attitudes in practice) that would impede the accommodation of change, or else would  impede its sustainability. | Residential aged care facility | Mixed methods | TeamSTEPPS (teamwork training system) intervention | Context is defined in relation to 3 major elements; culture, leadership and performance evaluation.  (Definition based on PARiHS^6^) | Observational coding tool  Team diagnostic survey  Sub-elements of context informed data analysis of qualitative data |

| **Author, Year, Country** | **Aim** | **Setting** | **Study design** | **Implementation focus** | **Definition of context** | **Measure of context** |
| --- | --- | --- | --- | --- | --- | --- |
| Glidewell et al. (2013)  UK | 1) To identify perceived patient and professional (nurses and healthcare assistants) barriers to the uptake of SHC^[[37]](#footnote-37)^, using behavioural theory, and to use these data to identify intervention components to optimise care.  2) To explore whether the TDF^4^ developed to understand the implementation behaviour of professionals could be applied to patient implementation behaviour. | Hospitals and satellite centres | Qualitative (interviews) | SHC^37^ where hospital haemodialysis patients are supported by dialysis staff to become as involved as they wish in their own care. | Any circumstance of a person’s situation or environment that discourages or encourages the development of skills and abilities, independence, social competence, and adaptive behaviour.  (Definition based on the TDF^4^) | TDF^4^ used to inform the topic guide and data analysis. |

| **Author, Year, Country** | **Aim** | **Setting** | **Study design** | **Implementation focus** | **Definition of context** | **Measure of context** |
| --- | --- | --- | --- | --- | --- | --- |
| Glisson et al. (2008)  USA | 1) Confirm the factor validity of the measure of OSC^9^ in a national sample of mental health clinics and clinicians.  2) Describe the variation in clinician responses to the OSC^9^ and in the organisational social context profiles that distinguish mental health clinics nationwide.  3) Assess the relationships that link dimensions of mental health clinic-level culture and climate profiles to clinician-level work attitudes | Community mental health clinics | Quantitative (survey development CFA) | Adoption of ChildSTEPs^[[38]](#footnote-38)^ | Organisational culture theory identifies culture and climate as central constructs in organisational social context. Climate refers to ‘‘the way people perceive their work environment’’ and culture refers to ‘‘the way things are done in the organisation’’ | OSC^9^ measure |
| Greenhalgh et al. (2008)  UK | To explore the introduction of a centrally stored, shared electronic patient record (the SCR^[[39]](#footnote-39)^) in England and draw wider lessons about the implementation of large-scale information technology projects in health care. | Each site consisted of a  primary care trust, participating general practices, and  one or more linked unscheduled care setting (such as an  ED^36^, walk-in centre, out of hours service) | Mixed methods | SCR^39^ | Context defined in relation to shifting political, economic, technological environments. | Adapted diffusion of innovation model guided data collection |

| **Author, Year, Country** | **Aim** | **Setting** | **Study design** | **Implementation focus** | **Definition of context** | **Measure of context** |
| --- | --- | --- | --- | --- | --- | --- |
| Griffin et al. (2017)  UK | To explore in depth the impacts and outcomes of visiting on a variety of healthcare organisations, focussing on the IVVs^[[40]](#footnote-40)^ undertaken in England as part of NHS England’s quality assurance of medical revalidation. | Hospital-sample of secondary care providers | Qualitative (interviews, observations, document analysis) | Independent verification visits | (1) the details of the setting; (2) the environment (the culture, the readiness and capacity for change) and (3) leadership (including effective teamwork and systems at the strategic and operational levels of the organisation).  (Definition based on MUSIQ^24^ framework) | MUSIQ^24^ framework informed data collection (interview guide) and analysis |
| Guerrero et al.  (2015)  USA | To identify the relationship between promising organisational factors and the uptake of PC-MH^[[41]](#footnote-41)^ services in the VHA^[[42]](#footnote-42)^ system. | VHA^42^ clinics | Quantitative (cross sectional survey) | Implementation of mental health care in primary care settings (co-located care) | Clinic stress, flexibility in decision making, and participatory decision making. | VHA^42^ Clinical Practice Organisational Survey |

| **Author, Year, Country** | **Aim** | **Setting** | **Study design** | **Implementation focus** | **Definition of context** | **Measure of context** |
| --- | --- | --- | --- | --- | --- | --- |
| Hansen et al. (2011)  Denmark | To draw attention to possible contextual dimensions that need to be considered when discussing, validating and acting on  conclusions from quantitative intervention studies. | Cancer rehabilitation centre | Qualitative (observations, interviews and written evaluations of interventions) | Cancer rehabilitation programme | Contextual dimensions include human interactions, the organisation of the intervention, the staff, the timing, the physical surroundings or the general atmosphere. The term context comes from Latin contexus, which means ‘to join together’ or ‘to compose’. Context is an act of composition, making  connections, of weaving together parts of language, behaviour and surroundings into meaningfulness. Context is not to be seen as a fixed, outer reality. Analytically, context can be understood as a kind of frame that becomes created and recreated around an event. Context involves a fundamental juxtaposition of two entities; the event being analysed and a field of actions, actors and surroundings within which that event is embedded | Observations used to collect data related to context  Conceptual framework proposed by Duranti and Goodwin guided data analysis |

| **Author, Year, Country** | **Aim** | **Setting** | **Study design** | **Implementation focus** | **Definition of context** | **Measure of context** |
| --- | --- | --- | --- | --- | --- | --- |
| Higgins et al. (2015)  UK | To investigate how organisational context facilitates or hinders interventions intended to manage LTSA^[[43]](#footnote-43)^, in  order to provide evidence for enabling and sustaining effective management approaches in large public sector organisations. | Health and Social care Trusts | Qualitative (interviews, observations, document analysis) | Initiatives to manage LTSA^43^ | Organisation size, absence culture, organisational change and job demands organisational structure, cultural mores, economic capacity, and the interpretations of the individuals involved | Developed programme theories from the literature informed data collection and realist principles guided analysis. |
| Hill et al. (2017)  USA | 1) To demonstrate value of utilising PARIHS^6^ retrospectively and prospectively.  2) To describe the process used to assess PARIHS^6^ constructs and sub-elements.  3) To report the extent of implementation. | VA^[[44]](#footnote-44)^ hospitals | Qualitative (case study) | Study1: implementation of MRSA guidelines  Study2: implementation of My HealtheVet in the Spinal Cord Injury and Disorder System of Care | Leadership: High level, mid-level, and front-line supervisors or team leaders with direct/indirect influence on implementation  Culture: Local values and beliefs regarding local approaches to a particular behaviour or clinical practice  Measurement: Existing systems or processes for monitoring group or individual-level performance related to a particular behaviour or clinical practice  (Definition based on PARiHS^6^) | PARiHS^6^ constructs and sub-elements guided the analysis of two previous studies |

| **Author, Year, Country** | **Aim** | **Setting** | **Study design** | **Implementation focus** | **Definition of context** | **Measure of context** |
| --- | --- | --- | --- | --- | --- | --- |
| Hoffman & Rodriguez (2015)  USA | To assess the associations between FRIs^[[45]](#footnote-45)^ treated in the ED^36^ among older adults in California and contextual county-level physical, social, and economic characteristics, and to assess how county-level economic conditions are associated with FRIs^45^ when controlling for other county-level factors. | Hospital-ED^36^ | Quantitative (random effects logistic regression models) | Translation of falls prevention programs to entire communities | Availability of resources and ownership of resource allocation, capacity of the community, and integration into established structures | County Health Rankings’ county level standardised scores used to obtain information for economic conditions, the built environment, community safety, access to care, and obesity within the study. |
| Huijg et al. (2014)  Netherlands | To develop a questionnaire based on the 12-domain version of the TDF^4^ and to test the psychometric properties of this questionnaire on a sample of healthcare professionals. | Settings: unclear Sample: participants were physical therapists | Quantitative (survey development CFA) | Physical activity interventions | Social setting (e.g. norms and support) organisational context (e.g. capacity and resources). | TDF^4^ used to guide data collection and analysis. |

| **Author, Year, Country** | **Aim** | **Setting** | **Study design** | **Implementation focus** | **Definition of context** | **Measure of context** |
| --- | --- | --- | --- | --- | --- | --- |
| Iribarren et al. (2015)  Argentina | To understand implementation issues encountered during pilot-testing of a mHealth intervention, and to identify system improvements that will inform future implementation in a larger-scale trial. | Public pulmonary-specialised hospital | Qualitative (descriptive observational) | TextTB, an interactive text-based intervention to promote adherence with TB medications | Contest comprised of 3 factors: structure, process, outcome.  Structure:  Requirements for sustainability: costs, management, and equipment needs, cost of the intervention, organisational/technical support for management and equipment, management (e.g., team required)  Process:  Altered practice and delivery of service, workflow changes and monitoring, aspects of intervention (i.e., how it fits)  Outcome:  Global effect: lessons learned, potential application to other settings  (Definition based on the sociotechnical framework) | The sociotechnical framework used to guide data collection and analysis |

| **Author, Year, Country** | **Aim** | **Setting** | **Study design** | **Implementation focus** | **Definition of context** | **Measure of context** |
| --- | --- | --- | --- | --- | --- | --- |
| Kramer et al. (2017)  USA | To measure the organisational culture and climate determinants previously linked to implementation of EBPs^8^ in behavioural health in primary care settings | Primary care FQHCs^[[46]](#footnote-46)^ | Mixed methods | Evidence based practice to treat  1) mood disorders (screening, diagnostic evaluation, telepsychiatry consultation, care management, and online peer support and counselling)  2) alcohol use disorders (including screening,  brief intervention, and referral to treatment as needed) | Organisational climate is conceptualised as individual and group perceptions of how the work environment affects the well- being of the organisation’s members.  Organisational culture is conceptualized as how the work is done in the organisation based on worker expectations. | OSC^9^ survey  OSC^9^ constructs used to develop interview guide  OSC^9^ sub-level constructs were used to guide data analysis |
| Lemmens et al.  (2009)  Netherlands | To investigate to what extent primary care professionals are able to change their processes for delivering care to COPD^[[47]](#footnote-47)^ patients, and  what professional  and organisational factors are associated with the degree of process implementation. | Primary care | Quantitative  (quasi-experimental design) | COPD^47^ management programme | Culture, organisational commitment to quality improvement and climate.  (Definition based on the framework developed based on the theoretical approaches by Cretinet al. and Linet al.) | Culture was assessed using the competing values framework  Commitment to quality improvement was measured by an overall average score on quality improvement questions by the European foundation for quality management scale  adapted for primary care  Climate was measured as  the average of four questions assessing expected collegial responses to initiating behaviours and performing tasks related to COPD management |

| **Author, Year, Country** | **Aim** | **Setting** | **Study design** | **Implementation focus** | **Definition of context** | **Measure of context** |
| --- | --- | --- | --- | --- | --- | --- |
| Menon et al. (2014)  USA | To identify contextual factors associated with facility-level variation in missed test results within the VA^44^ health system. | VA^44^ health facilities | Mixed methods | Electronic Health Record alert system | Organisational policies, procedures and culture, workflow and communication, content, hardware and software, user interface, measurement and monitoring and external rules and regulations.  (Definition based on the sociotechnical framework) | Survey and interview guide informed by the eight-dimensional sociotechnical model. |
| Murdoch (2016)  UK | To describe a conceptual framework for analysing the relationship between intervention and context of delivery at different levels of implementation. | Primary care | Qualitative (retrospective evaluation) | Telephone triage programme | Context includes anything external to the intervention which impedes or strengthens its effects. | Conceptual framework guided the analysis  1. Macro: Broader discourses, policies in play during  trial implementation, infra-structural relations;  2. Meso: Institutional, network relations, histories of  relationships and interaction prior to implementing the intervention;  3. Micro non-linguistic: Types of activity participants are engaged in and interactional arrangements of intervention delivery; and  4. Micro linguistic and non-linguistic: Acts (specific  actions within activities) and utterances involved in  intervention delivery. |

| **Author, Year, Country** | **Aim** | **Setting** | **Study design** | **Implementation focus** | **Definition of context** | **Measure of context** |
| --- | --- | --- | --- | --- | --- | --- |
| Naik et al. (2015)  USA | To present results from a formative evaluation of the partnership-building process between research teams and non-academic, clinical practice settings, integration of intervention components into multiple primary care clinics and training of existing primary care staff to serve as intervention providers of a telehealth intervention targeting rural patients with uncontrolled diabetes and depression. | Primary care team at a large VA^44^ medical centre | Qualitative (observations and document analysis) | Telehealth intervention to improve diabetes and depression outcomes. | The context element assesses implementation factors within the prevailing organisational culture; the nature of relationships between primary care leadership and individual clinicians and the current capacity for monitoring routine care processes.  (Definition based on PARiHS^6^) | PARiHS^6^ framework guided data collection and analysis |

| **Author, Year, Country** | **Aim** | **Setting** | **Study design** | **Implementation focus** | **Definition of context** | **Measure of context** |
| --- | --- | --- | --- | --- | --- | --- |
| Obrecht et al. (2014)  USA | 1) Describe nurses’ perceptions of evidence-based practice, including (a) the strength of the evidence (research evidence, clinical experiences, and patient preferences) for the Wong-Baker FACES Pain Scale and FPS-R^[[48]](#footnote-48)^ before and after the implementation and  (b) the quality of the context (culture, leadership, and evaluation) of the practice environment.  2) As part of the EBP^8^ change, compare nurses’ perceptions of EBP^8^ (strength of the evidence) and nurses’ perceptions of barriers to research utilisation before and after implementation of FPS-R^48^. | Hospital-paediatric medical centre | Quantitative (cross-sectional survey) | Education program on the introduction of a pain assessment instrument FPS-R^48^ | The quality and characteristics of the environment where the implementation of evidence occurs.  When the quality of the context is high, care delivery is patient centred, support exists for learning and innovation, and  decision making is decentralized. | Perceptions of Evidence-Based Practice Scale  BARRIERS scale |

| **Author, Year, Country** | **Aim** | **Setting** | **Study design** | **Implementation focus** | **Definition of context** | **Measure of context** |
| --- | --- | --- | --- | --- | --- | --- |
| Padwa et al. (2016)  USA | 1) To measure integrated behavioural care capacity using an evaluation tool among a small sample of primary care clinics, and to observe how measures of integrated care capacity shift over time among a subsample of clinics for which longitudinal data were available.  2) To describe how various outer and inner contextual factors promote or inhibit the development of integrated care capacity in primary care clinics. | Primary care | Mixed methods | Integrated behavioural health protocols | The outer contextual factors within the conceptual model include the socio-political context (e.g. legislation, policies, administrative costs), funding (e.g. continuity of funding, support tied to federal and state policies, contracting arrangements), client advocacy (e.g. consumer organisations, lawsuits), inter-organisational networks (e.g. organisational linkages, professional organisations, information sharing), leadership (e.g. developing readiness for innovation, developing systems with a learning orientation likely to facilitate implementation), and intervention developer engagement in implementation.  The model's inner contextual factors include organisational characteristics (e.g. absorptive capacity, structure, knowledge/skills/expertise), individual adopter characteristics (e.g. values, goals, perceived need for change), leadership (e.g. taking ownership of implementation, championing implementation), innovation-values fit  (e.g. how well new practices fit within the structure and ideology of the organisation), fidelity monitoring/support (e.g. role clarity when implementing new practices, supportive coaching), and staffing (e.g. staff selection criteria, staff selection procedures).  (Definition based on EPIS^33^ model) | Outer contextual and inner contextual factors from the Conceptual Model of Evidence-Based Practice Implementation in Public Service Sectors were used as codes during data analysis |

| **Author, Year, Country** | **Aim** | **Setting** | **Study design** | **Implementation focus** | **Definition of context** | **Measure of context** |
| --- | --- | --- | --- | --- | --- | --- |
| Prashanth et al. (2014)  India | To understand how capacity-building of health managers translates into improved performance with respect to their planning and  supervision in Tumkur district of Karnataka state in southern India. | District health system | Qualitative (document review, interviews, observations) | Capacity building intervention | Actors or other factors that occur in the setting where the intervention/policy was implemented, that occur independent of the  intervention/policy and affect the implementation of the intervention/policy. | To identify contextual factors, government reports  and program documents related to performance of district health services were reviewed and interview transcripts with participants of the intervention, co-workers of higher levels in the system were analysed using realist principles. |
| Presseau et al. (2017)  Canada | To investigate the barriers and enablers to physicians prescribing and nurses setting IDT^[[49]](#footnote-49)^ to inform a strategy to optimise the delivery of IDT^49^ in multiple haemodialysis centres that will be randomly allocated to the intervention arm of the MyTEMP trial. | Haemodialysis centres | Qualitative (interviews) | Individualised temperature-reduced haemodialysis | Physical location in which the behaviour takes place; in the dialysis centre.  (definition based on TDF^4^) | TDF^4^ informed the interview guides and data analysis |

| **Author, Year, Country** | **Aim** | **Setting** | **Study design** | **Implementation focus** | **Definition of context** | **Measure of context** |
| --- | --- | --- | --- | --- | --- | --- |
| Rabbani et al. (2011)  Pakistan | To explore contextual perspectives in relation to opportunities and challenges involved in BSC^[[50]](#footnote-50)^ implementation in  one hospital in Pakistan. | Hospital units | Mixed methods | Balanced Score Card- a management tool to improve team performance | Contextual factors influencing efforts towards achieving goals include presence of a participatory culture, employee commitment and  competence, technological resources, autonomy, degree of harmony between unit leader and employees, positive attitude towards the intervention being introduced, and supportive leadership.  Economic, political, and social factors at macro  level constitute the external context.  The internal context is characterised by organisational culture, leadership, human and financial resources, and type of healthcare setting | Pettigrew and Whipp's theoretical framework guided data collection and analysis (sub-elements of framework-context, content, process)  Validated questionnaire based on the competing values framework used to articulate cultural types  Participant observations used for an understanding of contextual sensitivity. |

| **Author, Year, Country** | **Aim** | **Setting** | **Study design** | **Implementation focus** | **Definition of context** | **Measure of context** |
| --- | --- | --- | --- | --- | --- | --- |
| Rodríguez & Peterson (2016)  Honduras | To conduct a retrospective review of a CHW^[[51]](#footnote-51)^ program using the generic logic model for CHW^51^ performance and identify the factors contributing to the program’s success in improving health outcomes. | Community | Qualitative (document review and interviews) | Integrated Child Health Program in the Community (AIN-C) (nutrition program). | National, political, economy, and community characteristics. | A logic model for community health worker performance that incorporates multiple dimensions (health system, community factors) guided data collection and analysis |
| Rotteau et al. (2015)  Canada | To describe the hospital-based implementation teams’ experiences during the program implementation, and the team’s perceptions of the key factors that influenced the program’s success or failure. | Hospital- ED^36^ | Qualitative (interviews) | ED^36^ process improvement program to reduce ED^36^ length of stay. | Four key categories of contextual factors associated with successful QI^[[52]](#footnote-52)^ implementation were identified: structural, political, emotional,  and cultural. | Interview guide addressed barriers and enablers to program implementation as well as perceptions of organisational factors related to achieving improved hospital and ED^36^ performance |
| Smith et al. (2018)  UK | To explore the barriers and facilitators of relocating patients from a specialised homeless health centre to mainstream general practice from the perspectives of formerly homeless patients and staff involved in their care | Primary care | Qualitative (interviews) | Relocation of formerly homeless patients eligible to transfer from SHHC^[[53]](#footnote-53)^ to mainstream general practices. | Any circumstance of a person’s situation or environment that discourages or encourages the development of skills and abilities, independence, social competence, and adaptive behaviour.  (Definition based of the TDF^4^) | TDF^4^ informed the interview guide and analysis |

| **Author, Year, Country** | **Aim** | **Setting** | **Study design** | **Implementation focus** | **Definition of context** | **Measure of context** |
| --- | --- | --- | --- | --- | --- | --- |
| Spitzer-Shohat et al. (2018)  Israel | To better understand the sources of variation in relation to clinic outcomes to identify "what works in which circumstance and for whom". | Primary care clinics | Mixed methods | Programme aimed at reducing disparities in socially disadvantaged groups | Contextual drivers; clinic team perceptions on possessing the necessary resources and skills to succeed, as well as relationships among team members and with their superior managerial units | Contextual characteristics such as clinic size and SES indicator score rating all residential areas in Israel from 1-low SES to 10 high SES were obtained.  Contextual drivers outlined in definition examined during interviews.  The characteristics of the clinics’ patient populations (outer context) as well as the clinics’ teams (inner context) were also examined. |
| Vanderkruik & McPherson (2017)  USA | To present work on designing a new contextual factors framework that was applied to several national public health initiatives and utilised as a tool for multiple purposes, including guiding evaluation, implementation, and reporting. | Public health services | Qualitative  (retrospective evaluation) | 3 public health interventions focused on sickle cell disease, breastfeeding promotion, and prevention of infant mortality. | Contextual factors can include anything external to the intervention (e.g., media and/or policy relevant to intervention topic, project  personnel) that might facilitate or hinder its level of success. | The developed Contextual Factors Framework guided data collection and analysis |

| **Author, Year, Country** | **Aim** | **Setting** | **Study design** | **Implementation focus** | **Definition of context** | **Measure of context** |
| --- | --- | --- | --- | --- | --- | --- |
| VanDevanter et al. (2017)  Vietnam | 1) To identify factors that may influence guideline implementation.  2) To inform further modifications to the intervention that may be necessary to translate a model of care delivery from a HIC^[[54]](#footnote-54)^ to the local context of a LMIC^[[55]](#footnote-55)^. | Community health centres | Qualitative (interviews) | Tobacco use treatment guidelines | Outer setting (e.g., perceived need for services for tobacco cessation in the community, role of the Ministry of Health policies in driving which services were implemented in these settings).  Inner setting (e.g., perceptions about leadership engagement, relative priority of tobacco use, compatibility of the proposed intervention,  and tobacco use treatment in general, with current workflows and staffing resources, experiences with various health care programs run by their respective Community Health Centres, and their reports of organisational factors affecting the implementation of past public health programs)  (Definition based on the CFIR^22^) | CFIR^22^ informed interview guide and data analysis |

| **Author, Year, Country** | **Aim** | **Setting** | **Study design** | **Implementation focus** | **Definition of context** | **Measure of context** |
| --- | --- | --- | --- | --- | --- | --- |
| Ware et al. (2018)  Canada | To evaluate the implementation of the Medley program by answering two research questions  (1) To what extent was the Medly program successfully implemented?  (2) What were the barriers and facilitators to implementing Medly program? | Heart failure clinic | Qualitative (longitudinal single case study-interviews) | Medly, a mobile phone based telemonitoring program | Outer setting (e.g. patient needs and resources, external policy and incentives).  Inner setting (e.g. networks and communication, implementation climate, readiness for implementation).  (Definition based on CFIR^22^) | CFIR^22^ informed the interview guide and guided analysis |
| Williams et al. (2016)  UK | 1)To identify the ways in which different intermediaries  influence practice.  2)To understand the context within which intermediaries  operate.  3)To develop CMO^15^ configurations that explain the relationship between specific mechanisms and conditions  and how this leads to change or particular outcomes.  4)To build and refine the context–mechanism–outcome  configurations through data collection. | Hospital | Qualitative  (case study-interviews and observations) | Role of intermediaries in implementing an infection prevention and control strategy. | Context is described as conditions which influence the success or failure of different interventions or programmes.  A range of factors can influence the success of implementing best practice in infection prevention and control, including the nature of leadership, managerial support, public reporting, structures, team stability, morale, workload and staffing | Realist principles informed data collection and analysis |
| Yamada et al. (2017)  Canada | To determine the influence of organisational context in moderating the effect of research use and pain outcomes. | Paediatric hospitals | Quantitative (cross-sectional survey) | Knowledge translation intervention- EPIQ^[[56]](#footnote-56)^ | Organisational contextual factors (i.e. work environment factors such as leadership, interactions, resources) | ACT^3^ |

| **Author, Year, Country** | **Aim** | **Setting** | **Study design** | **Implementation focus** | **Definition of context** | **Measure of context** |
| --- | --- | --- | --- | --- | --- | --- |
| Yamada et al. (2018)  Canada | To use a theory-based approach to develop an in-depth understanding of;  1) the perceived assumptions underpinning the pathway intervention from the perspectives of the pathway intervention developers and  2) the perceived barriers and enablers to use of the pathway indicated by: a) primary care physicians’ prescribing practices of controller medications; b) allied healthcare professionals providing asthma education to families; and c) parents adhering with their child’s treatment plan | Primary care | Qualitative (interviews) | Paediatric asthma management program (asthma clinical pathway and asthma education module). | Any circumstance of a person’s situation or environment that discourages or encourages the development of skills and abilities, independence, social competence, and adaptive behaviour.  (Definition based on the TDF^4^) | TDF^4^ informed the interview guides and data analysis |
| Yip et al. (2016)  USA | To describe the contextual factors that encourage or impede the implementation processes of a research-tested program. | Community health centres | Qualitative (case study-interviews) | Colorectal cancer screening program | The availability of resources and expertise, the presence of “champions” and leadership and the varying organisational culture in diverse health care settings that shape implementation. | RE-AIM^[[57]](#footnote-57)^ framework informed the interview schedule and data analysis. |

1. Electronic Medical Records [↑](#footnote-ref-1)
2. Paediatric Early Warning Score [↑](#footnote-ref-2)
3. Alberta Context Tool [↑](#footnote-ref-3)
4. Theoretical Domains Framework [↑](#footnote-ref-4)
5. Empowering Patients in Chronic Care [↑](#footnote-ref-5)
6. Promoting Action on Research Implementation in Health Services [↑](#footnote-ref-6)
7. Randomised Controlled Trial [↑](#footnote-ref-7)
8. Evidence Based Practice [↑](#footnote-ref-8)
9. Organisational Social Context [↑](#footnote-ref-9)
10. Implementation Climate Scale [↑](#footnote-ref-10)
11. Implementation Leadership Scale [↑](#footnote-ref-11)
12. Emergency Obstetric and Neonatal Care [↑](#footnote-ref-12)
13. Normalisation Process Theory [↑](#footnote-ref-13)
14. Sundere Liv iSocial psykiatrien (Healthier living in community mental health services) [↑](#footnote-ref-14)
15. Context-Mechanisms-Outcomes [↑](#footnote-ref-15)
16. Team Check-up Tool [↑](#footnote-ref-16)
17. Practice Environment Scale-Nursing Work Index [↑](#footnote-ref-17)
18. Team Functioning Survey [↑](#footnote-ref-18)
19. Keeping Childbirth Natural and Dynamic [↑](#footnote-ref-19)
20. Electronic Health Records [↑](#footnote-ref-20)
21. Allied Health Progress Note [↑](#footnote-ref-21)
22. Consolidated Framework for Implementation Research [↑](#footnote-ref-22)
23. Organisational Readiness to Change Assessment [↑](#footnote-ref-23)
24. Model for Understanding Success In Quality [↑](#footnote-ref-24)
25. Organisational Climate Measure [↑](#footnote-ref-25)
26. Uniform Patient Fee Schedule [↑](#footnote-ref-26)
27. Patients’ Rights Charter [↑](#footnote-ref-27)
28. Peripheral Venous Catheters [↑](#footnote-ref-28)
29. Electronic Patient Record [↑](#footnote-ref-29)
30. Primary Care Providers [↑](#footnote-ref-30)
31. Training and Education for the Advancement of Children's Health [↑](#footnote-ref-31)
32. Mental Health [↑](#footnote-ref-32)
33. Exploration, Preparation, Implementation, Sustainment [↑](#footnote-ref-33)
34. Integrated Knowledge Translation [↑](#footnote-ref-34)
35. Computerised Provider Order Entry [↑](#footnote-ref-35)
36. Emergency Department [↑](#footnote-ref-36)
37. Shared Haemodialysis Care [↑](#footnote-ref-37)
38. Child Systems and Treatment Enhancement Projects [↑](#footnote-ref-38)
39. Summary Care Record [↑](#footnote-ref-39)
40. Independent Verification Visits [↑](#footnote-ref-40)
41. Primary Care-Mental Health [↑](#footnote-ref-41)
42. Veteran Health Administration [↑](#footnote-ref-42)
43. Long-Term Sickness Absence [↑](#footnote-ref-43)
44. Veteran Affairs [↑](#footnote-ref-44)
45. Fall-Related Injuries [↑](#footnote-ref-45)
46. Federally Qualified Health Centres [↑](#footnote-ref-46)
47. Chronic Obstructive Pulmonary Disease [↑](#footnote-ref-47)
48. Faces Pain Scale-Revised [↑](#footnote-ref-48)
49. Individualised Dialysate Temperatures [↑](#footnote-ref-49)
50. Balanced Scorecard [↑](#footnote-ref-50)
51. Community Health Worker [↑](#footnote-ref-51)
52. Quality Improvement [↑](#footnote-ref-52)
53. Specialist Homeless Healthcare Centres [↑](#footnote-ref-53)
54. High-Income Country [↑](#footnote-ref-54)
55. Low-Middle Income Country [↑](#footnote-ref-55)
56. Evidence-based Practice for Improving Quality [↑](#footnote-ref-56)
57. Reach Effectiveness Adoption Implementation [↑](#footnote-ref-57)
